# Epithelial NLRP3 drives silica-induced lung injury and fibrosis through IL-18 and pro-fibrotic neutrophil recruitment

**DOI:** 10.1186/s12989-026-00682-9

**Published:** 2026-05-03

**Authors:** Maggie Lam, Kristian T. Barry, Christopher J. Hodges, Alison C. West, Christopher M. Harpur, Ashley Mansell, Michelle D. Tate

**Affiliations:** 1 Centre for Innate Immunity and Infectious Disease, Hudson Institute of Medical Research, Clayton, VIC 3168 Australia; 2 Department of Molecular Translational Sciences, Monash University, Clayton, VIC 3186 Australia; 3 Department of Microbiology, Anatomy, Physiology, and Pharmacology, La Trobe University, Bundoora, VIC 3086 Australia

**Keywords:** Silicosis, NLRP3 inflammasome, Epithelial cells, IL-18, Neutrophils, Fibrosis, Conditional knockout

## Abstract

**Background:**

Silicosis is a progressive inflammatory and fibrotic lung disease with no effective treatments beyond symptom management. While global NLRP3 inflammasome deficiency attenuates silica-induced pathology, myeloid-specific *Nlrp3* deletion provides no protection, suggesting that other cellular sources drive disease. Given that epithelial cells directly encounter inhaled silica particles and express NLRP3, we investigated the functional contribution of epithelial *Nlrp3* to silicosis pathogenesis.

**Results:**

Using inducible epithelial-specific knockout models, we found that alveolar epithelial (*Sftpc*^+^) cells drive early caspase-1 activation and airway IL-18 production, while partially reducing tissue IL-1β maturation. Loss of alveolar epithelial *Nlrp3* limited recruitment of pro-fibrotic Siglec-F^+^ neutrophils, which expressed elevated fibrogenic mediators, and reduced airway neutrophil elastase levels. At day 14, *Nlrp3* deficiency reduced persistent Siglec-F^+^ neutrophils and broadly reduced airway inflammatory cytokines, accompanied by decreased lung damage, alveolitis, collagen deposition, fibrotic nodule expansion, and α-SMA expression, independent of detectable TGFβ changes. By day 28, during the chronic fibrotic phase, alveolar epithelial *Nlrp3* deficiency continued to confer protection, reducing persistent inflammation, collagen accumulation, and fibrotic nodule size. In parallel, deletion of *Nlrp3* in *Scgb1a1*⁺ bronchiolar epithelial cells reduced early inflammasome activation at day 3 and similarly decreased chronic lung inflammation, collagen deposition, and fibrotic nodule expansion, without affecting nodule number or cellularity at day 28.

**Conclusions:**

These findings establish epithelial *Nlrp3* as a critical driver of silica-induced fibrotic remodeling through IL-18 and pro-fibrotic neutrophil recruitment, independent of canonical TGFβ mechanisms. This epithelial-centric paradigm provides a rationale for precision therapeutic strategies targeting epithelial NLRP3.

**Supplementary Information:**

The online version contains supplementary material available at 10.1186/s12989-026-00682-9.

## Background

Silicosis is a progressive, irreversible fibrotic lung disease caused by inhalation of respirable crystalline silica particles. Despite decades of prevention efforts, cases are surging globally, driven by acute forms among engineered stone workers in Australia, China, Brazil, South Africa, and Spain [1]. This has prompted urgent legislative reforms, including Australia’s 2024 ban on silica-rich benchtops [2]. Between 1990 and 2019, global silicosis prevalence and incidence increased by 91.4% and 64.6%, respectively [3], underscoring persistent failures in occupational safety and the need for therapies beyond symptom management.

Although silicosis often develops after years of cumulative exposure, acute forms can manifest within weeks to months following high-level exposure [4, 5]. Inhaled silica particles deposit in alveoli and airways, where they are internalized by alveolar macrophages (AMs) and epithelial cells, triggering release of pro-inflammatory (IL-1β, IL-6, TNF) and pro-fibrotic (TGFβ) mediators that drive severe lung inflammation and progressive fibrosis [5, 6]. Persistent inflammation causes epithelial injury and dysregulates myeloid cell function, reducing clearance of apoptotic cells. As cells undergo death, they release additional inflammatory mediators such as damage-associated molecular patterns (DAMPs) [7, 8]. Silica particles and cellular debris accumulate within granulomatous nodules, which coalesce into larger lesions known as progressive massive fibrosis, leading to severe impairment of lung function [9].

At the molecular level, a central mediator of silica-induced inflammation is the NLRP3 inflammasome, which activates caspase-1 to process pro-IL-1β and pro-IL-18 and also induces pyroptotic cell death [7, 10]. Silica causes phagosomal destabilization and lysosomal rupture, releasing inflammatory mediators, reactive oxygen species, and DAMPs, which activate NLRP3 [11, 12]. Although NLRP3 is a well-established driver of silica-induced inflammation and fibrosis, its origin remains unresolved. Global *Nlrp3* deficiency in an acute in vivo model markedly attenuates disease severity, implicating this inflammasome as a key pathogenic mediator [13]. Yet, we recently described that deletion of *Nlrp3* specifically in myeloid cells did not confer protection [14], challenging the long-held view that alveolar macrophages are the primary source [7, 10]. This paradox suggests that alternative cellular sources of NLRP3 may initiate and sustain pathology.

With myeloid sources excluded, lung epithelial cells emerge as a plausible candidate for pathogenic NLRP3 activity. As the first barrier to inhaled particles, epithelial cells also express NLRP3 [15] and can activate the inflammasome in response to silica in vitro [16]. We previously demonstrated NLRP3 upregulation in vivo using an endogenous reporter mouse, identifying reporter-positive cells lining the airways and alveoli consistent with airway and alveolar epithelial cells based on anatomical location [13]. Because silica particles deposit throughout both alveolar and distal airway regions, and NLRP3 is upregulated in both epithelial populations in vivo, both compartments may contribute to disease initiation and progression. Unlike macrophages, which require NF-κB driven transcriptional induction of inflammasome substrates, epithelial cells constitutively store pro-IL-18 and can rapidly mature IL-18 upon caspase-1-dependent activation [17, 18]. However, the functional contribution of epithelial NLRP3 to silicosis pathogenesis remained undefined.

Here, we address this gap using inducible epithelial-specific *Nlrp3* knockout models targeting *Sftpc*^+^ and *Scgb1a1*^+^ compartments, enabling analysis of disease progression from acute to chronic phases (days 3–28). Alveolar epithelial *Nlrp3* deletion reduced caspase-1 activation, selectively impaired airway IL-18 production, and limited recruitment of pro-fibrotic Siglec-F^+^ neutrophils, attenuating progressive fibrotic remodeling and collagen deposition independently of detectable TGFβ changes. Bronchiolar epithelial *Nlrp3* deletion similarly reduced overall lung pathology, inflammation, and fibrosis, demonstrating broad epithelial contribution to disease progression. These findings resolve a longstanding mechanistic paradox and identify epithelial NLRP3 as a precision therapeutic target with implications for inhaled delivery strategies.

## Methods

### Animals

Wildtype, *Sftpc-*CreER^T2^ (*Sftpc*), *Sftpc-*CreER^T2^ x *Nlrp3*^fl/fl^, *Scgb1a1*-CreER™ (*Scgb1a1*), and *Scgb1a1*-CreER™ x *Nlrp3*^fl/fl^ mice on the C57BL/6J background (male and female, 6–14 weeks) were housed in specific pathogen-free conditions at the Monash Health Translational Precinct (Clayton, Victoria, Australia). *Nlrp3*^fl/fl^ mice were generated as previously described [14]. Transgenic mice expressing a tamoxifen-inducible Cre recombinase from the endogenous promoter/enhancer elements of the *Sftpc* [19] or *Scgb1a1* [20] locus were a kind gift from Brigid L. M. Hogan (Duke University).

To induce stable deletion of *Nlrp3* in *Sftpc*^+^ type II alveolar epithelial cells [19, 21, 22], *Sftpc*-CreER^T2^ x *Nlrp3*^fl/fl^ and *Sftpc*-CreER^T2^ mice were treated with tamoxifen (tmx; 0.2 mg/g body weight; Sigma, S3547) via intraperitoneal injection. Similarly, *Scgb1a1*-CreER™ x *Nlrp3*^fl/fl^ and *Scgb1a1*-CreER™ mice received the same treatment to excise *Nlrp3* in *Scgb1a1*^+^ non-ciliated bronchiolar Clara cells [19, 20, 23]. All mice received 3 tamoxifen treatments, 48 h apart, prior to silica exposure. Control mice received an equivalent volume of vehicle (veh; sesame oil, Sigma, S3547). Silica exposure occurred 72 h after the final tamoxifen dose to allow recombination.

All procedures were approved by the Hudson Animal Ethics Committee (Application MMCB/2021/18) in accordance with the Australian Code of Practice for the Care and Use of Animals for Scientific Purposes.

### Mouse model of silica-induced pulmonary disease

To model silica-induced lung injury, mice were lightly anaesthetized with isoflurane and intranasally instilled with 2 mg of crystalline silica (Min-U-Sil-5, U.S. Silica; > 99% purity and particle size of ≤ 5 μm) suspended in 50 µL phosphate buffered saline (PBS), following established protocols [24]. Control mice received PBS alone. Body weight was monitored 3 times a week. Mice were euthanized by intraperitoneal injection of sodium pentobarbital on day 3 for inflammatory endpoints and day 14 or 28 for analysis of fibrosis and tissue damage.

### Sample collection and processing

Bronchoalveolar lavage (BAL) was performed by flushing the lungs 3 times with 1 mL of PBS. BAL fluid was collected post-centrifugation for cytokine and protease analysis, while cell pellets were processed for flow cytometry. Lung tissues, post-BAL collection, were either snap-frozen in liquid nitrogen for protein analysis or fixed via inflation with 10% neutral buffered formalin (NBF) for histological evaluation. Mice were randomly allocated to experimental groups where possible.

### Immunoblot analysis of protein expression in lung tissues and BAL fluid

Lung tissues were homogenized in 5x SDS lysis buffer (250 mM Tris-HCl (pH 6.8), 10% (w/v) SDS, 20% (v/v) glycerol) supplemented with cOmplete™ Protease Inhibitor (Roche). Lung protein concentrations were quantified using the Bio-Rad DC Protein Assay. Protein in 1 mL of BAL fluid was concentrated using Strataclean resin (Agilent Technologies). Equal amounts of protein were resolved on 4–12% SDS-PAGE gels (Life Technologies), transferred to PVDF membranes (Merck Millipore), and probed with primary antibodies against NLRP3 (A41812012, AdipoGen Life Sciences), IL-1β (BAF401, R&D Systems), caspase-1 (clone Casper-1, AG-20B-0042-C100, AdipoGen Life Sciences), and α-tubulin (clone YL1/2; Abcam). Detection was performed using fluorescent secondary antibodies and imaged with the Bio-Rad ChemiDoc MP Imaging System.

### Quantification of cytokines and neutrophil elastase in BAL fluid

BAL fluid was analyzed for IL-1β and IL-18 using ELISA kits according to the manufacturer’s instructions (R&D Systems). Levels of IFNγ, IL-6, IL-10, IL-12p70, CCL2/MCP-1, and TNF were quantified by cytometric bead array (CBA; BD mouse inflammation kit, BD Biosciences). Neutrophil elastase protein levels were also measured via ELISA (R&D Systems) as per the manufacturer’s instructions, and activity was determined via incubation with substrate N-Methoxysuccinyl-Ala-Ala-Pro-Val p-nitroanilide (Sigma-Aldrich), as previously described [25].

### Flow cytometry analysis of BAL cells

BAL cells were pelleted, treated with red blood cell lysis buffer (Sigma Aldrich), and resuspended in FACS buffer (PBS supplemented with 2% (v/v) FBS and 2 mM EDTA). BAL cells were incubated with specific fluorescently labeled antibodies at 4°C for 30 min in the presence of Fc receptor-blocking monoclonal antibodies against CD16/32 (clone 93, Thermo Fisher Scientific) to limit nonspecific antibody binding. As previously described [12], BAL cells were stained with fluorochrome-conjugated antibodies as follows: CD11c (clone HL3, BD Biosciences), Ly6C (clone AL-21, BD Biosciences), CD64 (clone X54-5/7.1, BioLegend), CD3ε (clone 145-2C11, BioLegend), NK1.1 (clone PK136, BioLegend), Ly6G (clone 1A8, BD Biosciences), CD11b (clone M1/70, BioLegend), CD24 (clone M1/69, BioLegend), I-A^b^ (clone AF6-120.1, BD Biosciences), and Siglec-F (clone E50-2440, BD Biosciences) using the gating strategy outlined in Supplementary Figure S2. Viability was assessed using Zombie Aqua (BioLegend). In the indicated experiments, additional markers included CD69 (clone H1.2F3, BD Biosciences), CD206 (clone MR6F3, eBiosciences), and CD49b (clone DX5, BioLegend). For these experiments, Zombie NIR (BioLegend) was used to assess viability. Total live cells (viability dye^−^), neutrophils (Ly6G^+^ Ly6C^int^ SSC^lo^), natural killer (NK) cells (NK1.1^+^ CD3^−^), T cells (NK1.1^−^ CD3^+^), inflammatory macrophages (IM; Ly6G^−^ Ly6C^hi^), alveolar macrophages (AM; CD11c^+^ Siglec-F^+^ Ly6C^int^), dendritic cells (DCs; CD11c^+^ I-A^b+^), and eosinophils (CD24^+^ Siglec-F^+^ Ly6G^−^) were quantified by flow cytometry using an Aurora flow cytometer (Cytek Biosciences) and FlowJo™ 10 analysis software (BD Biosciences). Cell counts were determined using a standardized quantity of calibration particles/beads (ProSciTech) on a hemocytometer.

### Transcriptional analysis of airway neutrophils

Siglec-F^−^ and Siglec-F^+^ Ly6G^+^ neutrophils were flow-sorted from BAL samples using an Aurora flow cytometer (Cytek Biosciences). Total RNA was extracted with the RNeasy mini kit (QIAGEN), according to the manufacturer’s instructions. RNA was DNase-treated (Promega) and reverse transcribed to complementary DNA using Moloney murine leukemia virus reverse transcriptase (M-MLV; Promega), according to the manufacturer’s instructions. Real-time quantitative PCR was performed with Power SYBR Green chemistry (Life Technologies) on a QuantStudio 6 Flex (Applied Biosystems). The mRNA expression of target genes was normalized to the housekeeping gene *Gapdh*. The following primer sequences were utilized: *Il1b* Forward CAACCAACAAGTGATATTCTCCATG, Reverse GATCCACACTCTCCAGCTGCA; *Tnf* Forward ATGAGCACAGAAAGCATGATCCGC, Reverse CCAAAGTAGACCTGCCCGGACTC; *Tgfb1* Forward ACTGGAGTTGTACGGCAGTG, Reverse GGGGCTGATCCCGTTGATT; *Fgf2* Forward GGCTGCTGGCTTCTAAGTGT, Reverse GTCCAGGTCCCGTTTTGGAT; *Il1r2* Forward GTTTCTGCTTTCACCACTCCA, Reverse GAGTCCAATTTACTCCAGGTCAG ; *Il18rap* Forward ACCCAGAATTAGGAGCCCCC Reverse GGGGGCTCCTAATTCTGGGT; *Gapdh* Forward CATGGCCTTCCGTGTTCCTA, Reverse GCGGCACGTCAGATCCA.

### Histological assessment of lung pathology

Following fixation and inflation with 10% neutral buffered formalin (NBF), lung tissues were processed, paraffin embedded, and sectioned at 4 μm. All histological assessments were performed on Masson’s trichrome-stained sections. Fibrosis and alveolitis were semi-quantitatively scored using established criteria [13, 14]. Alveolitis was graded on a 0–5 scale based on peribronchial and perivascular inflammatory cell infiltration: 0 = no inflammation, 1 = very mild, 2 = mild, 3 = moderate, 4 = marked, and 5 = severe inflammation. Fibrosis was assessed using a modified Ashcroft scale (0–4): 0 = normal, 1 = minimal fibrous thickening of alveolar or bronchiolar walls, 2 = moderate thickening of walls without architectural distortion, 3 = increased fibrosis with formation of small fibrous masses or nodules and/or epithelial denudation, and 4 = severe structural distortion with large fibrous areas and obliteration of alveolar spaces. Five randomly selected fields per mouse were scored by three blinded, independent researchers, with samples corresponding to the least severe and most severe assigned scores of 0 and 4/5, respectively.

Quantitative morphometric analyses were also performed on trichrome-stained sections. Mean linear intercept (MLI) was calculated to estimate alveolar airspace enlargement by measuring the distance between alveolar walls [26]. Alveolar wall thickness was determined by measuring septal thickness across randomly selected regions.

Lung sections were viewed on an Olympus BX60 microscope and photographed at 20x, 10x or 4x magnification with an Olympus DP74 color camera using Olympus cellSens Dimension software. Masson’s trichrome staining intensity was quantified using ImageJ software (positive pixel intensity per FOV). In addition, silicotic nodule number, size/area, and cellularity were analyzed using Olympus cellSens Dimension [13].

### Immunohistochemical and immunofluorescent imaging of lung tissue sections

For alpha-smooth muscle actin (α-SMA) detection, longitudinal paraffin-embedded lung tissue sections (4 μm thickness) were dewaxed, rehydrated, and incubated in 3% hydrogen peroxide to quench endogenous peroxidase activity. Heat-induced antigen retrieval was performed using a citrate buffer (10 mM citrate, pH 6) at 100 °C for 6 min. Non-specific binding was blocked by incubating sections with CAS-Block Histochemical Reagent (Thermo Fisher Scientific) for 1 h at room temperature. Sections were then incubated overnight at 4°C with a primary antibody targeting alpha-smooth muscle actin (α-SMA, ab7817, Abcam), followed by a 2 h incubation with the appropriate secondary antibody at room temperature. Immunoreactivity was detected using diaminobenzidine (DAB) as the chromogen (Agilent Dako, K3469), and sections were counterstained with hematoxylin. Slides were mounted using D.P.X. mounting medium (Sigma Aldrich) and imaged at 20x magnification using an Olympus BX60 microscope and DP74 color camera. Five randomly selected fields of view (FOV) per mouse were captured, and DAB-positive staining (pixel count per FOV) was quantified using ImageJ software, as previously described [27].

For detection of TGFβ, separate sections were processed for immunofluorescence. Slides were dewaxed, rehydrated, and subjected to heat-induced antigen retrieval using 10 mM citrate buffer (pH 6) at 100°C for 6 min. After blocking with CAS-Block Histochemical Reagent for 1 h, sections were incubated overnight at 4°C with primary antibody against TGFβ (ab215715, Abcam). Slides were then washed with PBS containing 0.01% Tween 20 and incubated with fluorophore-conjugated secondary antibodies (Thermo Fisher) for 1 h at room temperature. Nuclei were stained with Hoechst 33,342 (Thermo Fisher), and slides were mounted using Fluorescence Mounting Medium (Agilent). Imaging was performed at 40x magnification using a Nikon AXR confocal microscope. Quantification of TGFβ expression was conducted using ImageJ software, with the % positive cells per FOV calculated across five randomly selected fields per mouse.

### Statistical analysis

Data were tested for normality and analyzed using GraphPad Prism Version 10 software (Graphstats Technologies, India). Student’s *t*-test (two-tailed, unpaired) was used when comparing two groups. When comparing three or more groups, a One-way analysis of variance (ANOVA) was used with a Tukey’s multiple comparisons post-hoc test. A *P* value of < 0.05 was considered statistically significant.

## Results

### Alveolar epithelial- *Nlrp3* contributes to silica-induced inflammasome activation

**Fig. 1:**
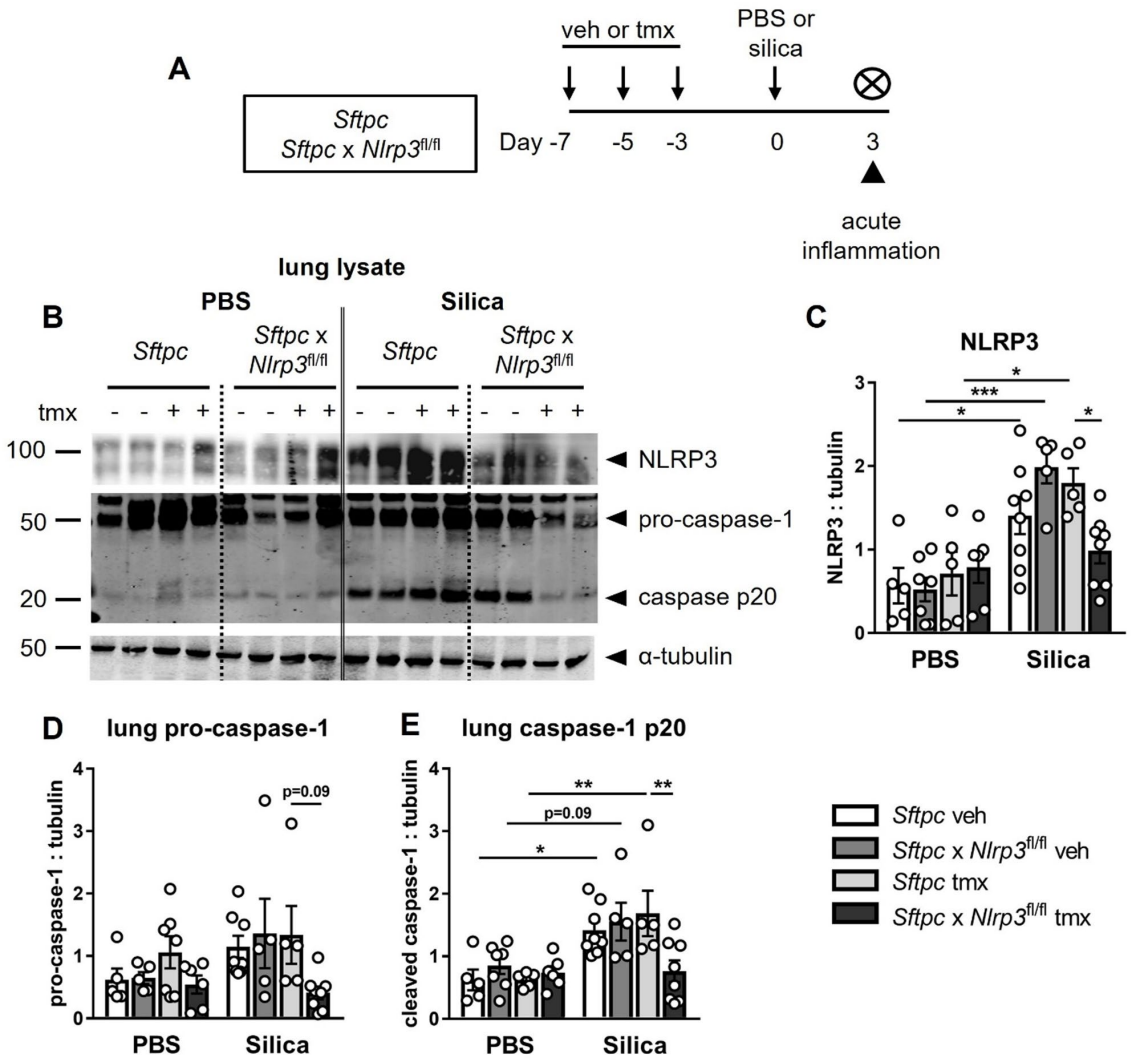
Alveolar epithelial-*Nlrp3* contributes to silica-induced inflammasome activation.** A** Experimental timeline for silica-induced lung injury in genetically modified mice. *Sftpc-*CreER^T2^ (*Sftpc*, control) and *Sftpc-*CreER^T2^ x *Nlrp3*^fl/fl^ (*Sftpc* x *Nlrp3*^fl/fl^, conditional knockout) mice received tamoxifen (tmx) or vehicle (veh) on days − 7, -5, and − 3 to induce Cre recombination. Mice were intranasally administered PBS or 2 mg of silica. Acute inflammation was assessed on day + 3. **B** Representative immunoblot of NLRP3, pro-caspase-1 (p45), cleaved caspase-1 (p20), and tubulin proteins in lung lysates. **C**–**E** Quantification of NLRP3 (**C**), pro-caspase-1 (p45; **D**), and cleaved caspase-1 (p20; **E**) relative to tubulin, pooled from 3 independent experiments. Data are presented as mean ± SEM, with each data point representing an individual animal. *N* = 5–8 per group. *P < 0.05, **P < 0.01, ***P < 0.001, One-way ANOVA with Tukey’s multiple comparisons test

To assess the contribution of alveolar epithelial cell-derived *Nlrp3* to silica-induced disease, *Nlrp3*^fl/fl^ mice were crossed with *Sftpc*-CreER^T2^ mice to enable cell-specific and stable deletion in alveolar type II epithelial cells [19, 21, 22]. To induce deletion of *Nlrp3*, *Sftpc*-CreER^T2^ and *Sftpc*-CreER^T2^ x *Nlrp3*^fl/fl^ mice were treated with tamoxifen (or vehicle as a control), followed by intranasal administration of silica (2 mg) or PBS [13, 14]. Mice were assessed at day 3 for inflammasome activation (Fig. 1A).

Immunoblot analysis revealed that silica exposure increased total NLRP3 expression (Fig. 1B, C) and cleaved caspase-1 (Fig. 1B, E) in the lung tissue and BAL from control mice (Supplementary Figure S1A, B). The mature p20 subunit of cleaved caspase-1, a marker of NLRP3 activation, was reduced in lung tissue from tamoxifen-treated *Sftpc*-CreER^T2^ x *Nlrp3*^fl/fl^ mice (Fig. 1B, E), whereas pro-caspase-1 levels trended lower (*p* = 0.09; Fig. 1D). In contrast, analysis of an equivalent volume of BAL fluid showed that the release of cleaved caspase-1 into the airways was not altered by alveolar epithelial *Nlrp3* deletion, suggesting that other cell types contribute to the increased caspase-1 activity (Supplementary Figure S1A, B).

Together, these findings demonstrate that alveolar epithelial NLRP3 is required for tissue-specific inflammasome activation in response to silica, while other cell types contribute to caspase-1 activity in the airway.

### Alveolar epithelial *Nlrp3* promotes silica-induced IL-18 production and contributes to IL-1β responses

**Fig. 2:**
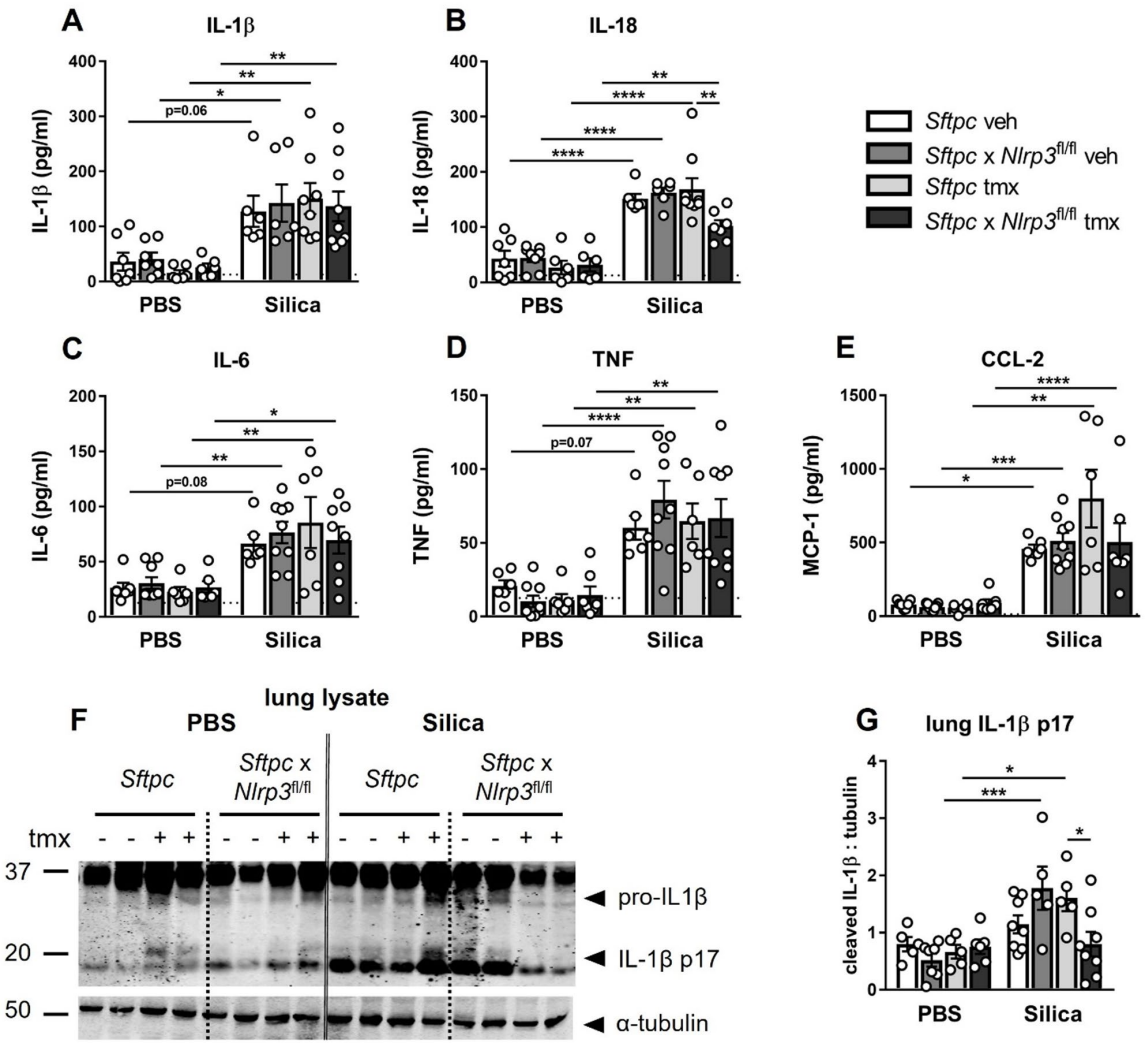
Alveolar epithelial *Nlrp3* selectively promotes IL-18 production and contributes to IL-1β responses following silica exposure. **A**–**G** *Sftpc*-CreER^T2^ (*Sftpc*, control) and *Sftpc*-CreER^T2^ x *Nlrp3*^fl/fl^ (*Sftpc* x *Nlrp3*^fl/fl^, conditional knockout) mice received tamoxifen (tmx) or vehicle (veh) on days − 7, -5, and − 3 to induce Cre recombination. Mice were subsequently intranasally administered PBS or 2 mg of silica. Analysis was performed on day + 3. Levels of (**A**) IL-1β and (**B**) IL-18 in BAL fluid quantified by ELISA. **C**–**E** Levels of IL-6, TNF, and CCL2/MCP-1 in BAL fluid quantified by CBA. **F** Immunoblot of lung tissue lysates for pro-IL1β (p31), mature IL-1β (p17), and tubulin protein. **G** Quantification of mature IL-1β (p17) relative to tubulin. **A**–**G** Data are presented as mean ± SEM, with each data point representing an individual animal. *N* = 5–8 per group. *P < 0.05, **P < 0.01, ***P < 0.001, ****P < 0.0001, One-way ANOVA with Tukey’s multiple comparisons test. Data are pooled from 3 independent experiments. The detection limit of each assay is noted as a dotted line

Given that alveolar epithelial *Nlrp3* contributes to caspase-1 activation (Fig. 1), we next assessed inflammasome-dependent cytokines at day 3 post-silica exposure. ELISA of BAL fluid showed that alveolar epithelial *Nlrp3* deletion selectively reduced IL-18, whereas IL-1β remained unchanged (Fig. 2A, B). Consistent with the selective early cytokine profile observed in global *Nlrp3*^−/−^ mice [13], other BAL cytokines measured by CBA (IL-6, TNF, and MCP-1/CCL2) were also unaffected at this timepoint (Fig. 2C–E). Immunoblot analysis of BAL fluid confirmed that the mature p17 form of IL-1β was unchanged by epithelial *Nlrp3* deletion (Supplementary Figure S1C, D). Additionally, IL-1β processing within lung tissue was examined. Immunoblot of post-BAL lung lysates revealed a significant reduction in mature IL-1β (p17) in alveolar epithelial *Nlrp3*-deficient mice (Fig. 2F, G), indicating that epithelial NLRP3 contributes to IL-1β maturation in the tissue compartment.

Collectively, these findings demonstrate that alveolar epithelial NLRP3 selectively regulates early inflammasome-dependent cytokines, driving IL-18 production in the airways and contributing to IL-1β maturation within lung tissue following silica exposure.

### Alveolar epithelial *Nlrp3* drives the recruitment, activation, and persistence of pro‑fibrotic neutrophils following silica exposure

**Fig. 3:**
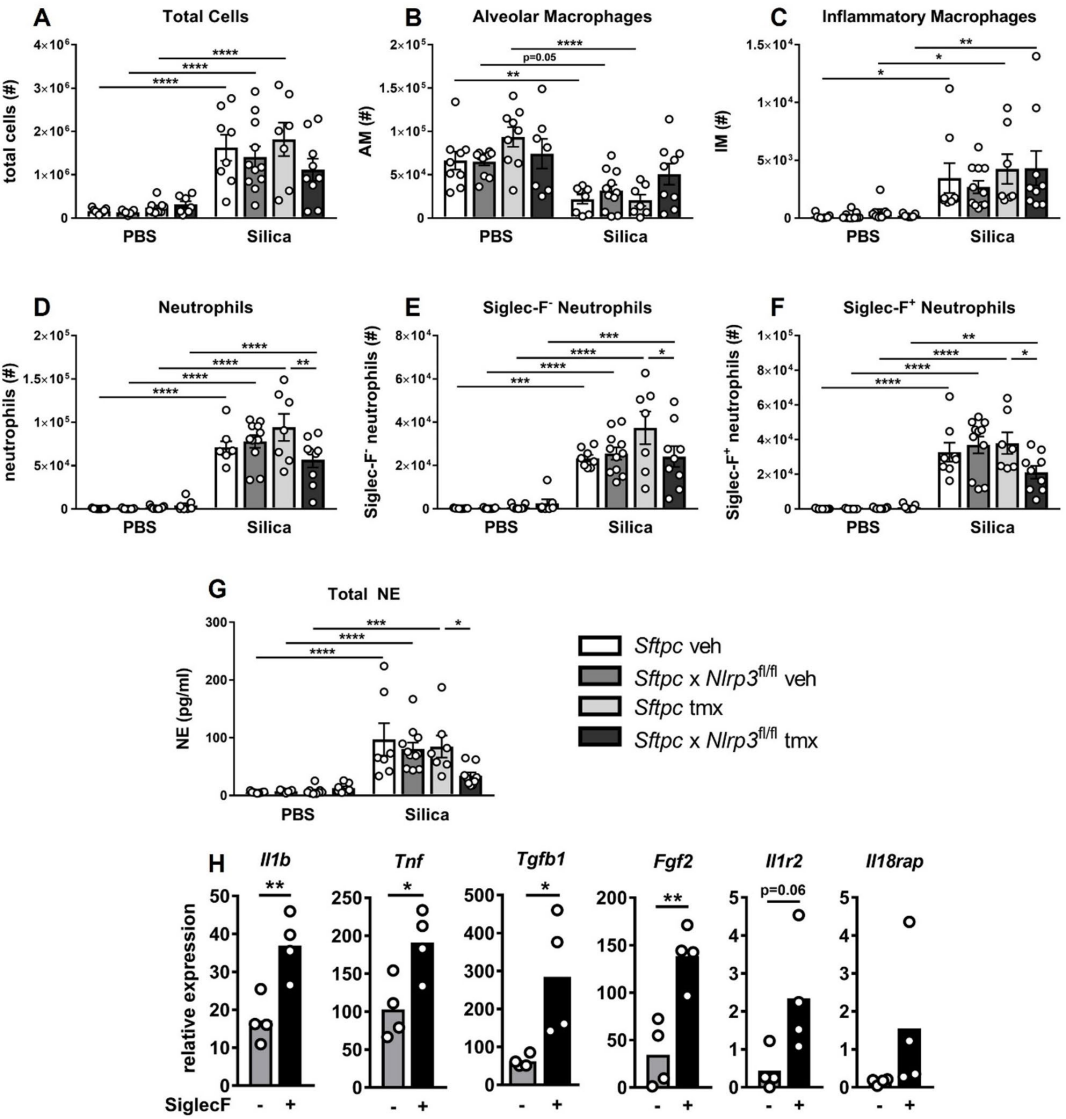
*Nlrp3* deficiency in alveolar epithelial cells limits infiltration of pro-fibrotic neutrophils following silica exposure. **A**–**H** *Sftpc*-CreER^T2^ (*Sftpc*, control) and *Sftpc*-CreER^T2^ x *Nlrp3*^fl/fl^ (*Sftpc* x *Nlrp3*^fl/fl^, conditional knockout) mice received tamoxifen (tmx) or vehicle (veh) on days − 7, -5, and − 3 to induce Cre recombination. Mice were subsequently intranasally administered PBS or 2 mg of silica. Analysis of BAL was performed on day + 3. Numbers (#) of live **A** total BAL cells, **B** alveolar macrophages (AMs), **C** Ly6C^hi^ inflammatory monocytes/macrophages (IMs), and **D** Ly6G^+^ neutrophils, determined by flow cytometry. Numbers (#) of live **E** Siglec-F^−^ and **F** Siglec-F^+^ neutrophil subsets. **G** Neutrophil elastase (NE) in BAL fluid. The assay detection limit was 12.5 pg/mL. **H** mRNA expression of *Il1b*,* Tnf*,* Tgfb1*, *Fgf2*,* Il1r2 and Il18rap* in flow-sorted Siglec-F^−^ and Siglec-F^+^ neutrophils isolated from the BAL of silica-exposed wildtype mice. Data are normalized to *Gapdh*. **A**–**H** Data are presented as mean ± SEM, with each data point representing an individual animal. *N* = 4–11 per group. *P < 0.05, **P < 0.01, ***P < 0.001, ****P < 0.0001, One-way ANOVA with Tukey’s multiple comparisons test. Data are pooled from 2–5 independent experiments

Given that IL-1β and IL-18 shape early innate responses [28], we next assessed whether alveolar epithelial *Nlrp3* influences airway immune cell composition at day 3 post-silica exposure. Flow cytometry was used to quantify total BAL cells and major immune subsets, including Ly6G^+^ neutrophils, Ly6C^hi^ inflammatory monocytes/macrophages (IMs), natural killer (NKs) cells, dendritic cells (DCs), eosinophils, T cells, and resident alveolar macrophages (AMs). Gating strategies shown in Supplementary Figure S2 were applied as previously described [13]. Silica exposure increased total BAL cellularity (Fig. 3A), driven predominately by the influx of IMs (Fig. 3C) and neutrophils (Fig. 3D). AM numbers decreased following silica exposure, consistent with known silica-induced cytotoxicity. Deletion of *Nlrp3* in alveolar epithelial cells significantly reduced neutrophil infiltration compared with silica-exposed controls (Fig. 3D), whereas IMs (Fig. 3C), AMs (Fig. 3B), and other innate and adaptive subsets (DCs, eosinophils, NK cells, and T cells) showed no significant differences (Supplementary Figure S3A).

To further characterize the neutrophil response, we examined conventional Siglec-F^−^ neutrophils and the pro-fibrotic Siglec-F^+^ subset [29]. Both populations were elevated following silica exposure, but alveolar epithelial *Nlrp3* deletion preferentially reduced the Siglec-F^+^ subset (Fig. 3E), indicating selective attenuation of early pro-fibrotic neutrophil recruitment. Consistent with reduced activation, neutrophil elastase, a granule-derived protease implicated in tissue remodeling and injury [30, 31], was significantly reduced in knockout mice.

Phenotypic profiling revealed that Siglec‑F⁺ neutrophils expressed higher levels of the activation‑associated markers CD69 and CD206 (Supplementary Figure S3E-F). Importantly, analysis of CD69^+^CD206^+^ double-positive neutrophils demonstrated that these activated cells represented a disproportionately larger fraction of the Siglec-F^+^ population (Supplementary Figure S3G), indicating preferential activation of this pro‑fibrotic subset during early silica-induced inflammation. Of note, AM activation was also assessed at day 3 by examining expression of MHC class II, CD11b, and CD64. Consistent with the absence of alterations in CCL2 at this early timepoint (Fig. 2E), AM activation did not differ between genotypes (data not shown).

We next examined the transcriptional programs of Siglec-F⁺ and Siglec-F⁻ neutrophils flow-sorted from wildtype mice at day 3. Siglec-F⁺ neutrophils exhibited elevated expression of activation- and pro-fibrotic-associated genes, including *Tgfb1*, *Fgf2*, *Tnf*, and *Il1b* (Fig. 3H). Notably, these cells also expressed *Il18rap* and *Il1r2* at the transcriptional level, suggestive of the capacity to respond to inflammasome-dependent cytokine signaling.

**Fig. 4:**
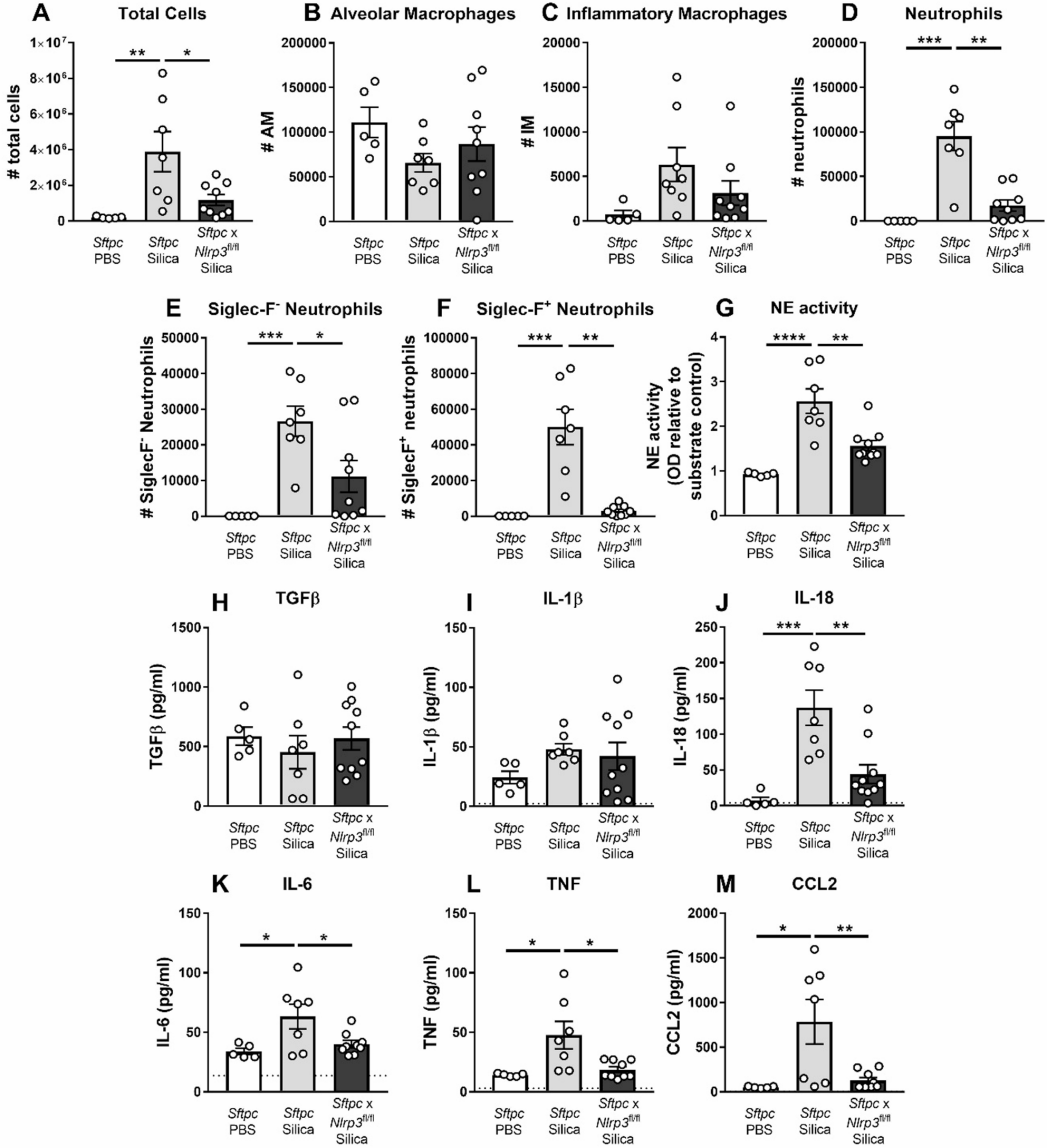
Alveolar epithelial *Nlrp3* regulates the persistence of pro‑fibrotic neutrophils and inflammatory cytokines during silica‑induced lung inflammation. **A**–**M** *Sftpc*-CreER^T2^ (*Sftpc*, control) and *Sftpc*-CreER^T2^ x *Nlrp3*^fl/fl^ (*Sftpc* x *Nlrp3*^fl/fl^, conditional knockout) mice received tamoxifen (tmx) or vehicle (veh) on days − 7, -5, and − 3 to induce Cre recombination. Mice were subsequently intranasally administered PBS or 2 mg of silica. Analysis of BAL was performed on day + 14. Numbers (#) of **A** total BAL cells, **B** alveolar macrophages (AMs), **C** Ly6C^hi^ inflammatory monocytes/macrophages (IMs), and **D** Ly6G^+^ neutrophils, determined by flow cytometry. Numbers (#) of live **E** Siglec-F^−^ and **F** Siglec-F^+^ neutrophil subsets. **G** Neutrophil elastase (NE) activity in BAL fluid (colorimetric assay; optical density; OD). **H** TGFβ, **I** IL-1β and **J** IL-18 in BAL fluid, determined by ELISA. **K**–**M** Levels of IL-6, TNF, and CCL2/MCP-1 in BAL fluid determined by CBA. The detection limit of each assay is noted as a dotted horizontal line. **A**–**M** Data are presented as mean ± SEM, with each data point representing an individual animal. *N* = 4–11 per group. *P < 0.05, **P < 0.01, ***P < 0.001, ****P < 0.0001, One-way ANOVA with Tukey’s multiple comparisons test. Data are pooled from 5 independent experiments

Given evidence that pro-fibrotic neutrophils can persist beyond the acute inflammatory phase [29, 32, 33], we assessed neutrophil populations at day 14. In control groups, Siglec-F⁺ neutrophils remained elevated (Fig. 4F), comprising ~ 50% of total neutrophils at day 14, consistent with sustained fibrotic inflammation. In contrast, alveolar epithelial *Nlrp3*-deficient mice showed a marked reduction in Siglec-F⁺ neutrophils, accompanied by decreased total neutrophil numbers (Fig. 4F), demonstrating that alveolar epithelial *Nlrp3* is required for both early recruitment and the persistence of pro-fibrotic neutrophils. AM and IM numbers remained unchanged by *Nlrp3* deletion (Fig. 4B-C).

Assessment of airway cytokines revealed a reduced inflammatory milieu in *Nlrp3*‑deficient mice at day 14. Silica‑exposed controls displayed elevated IL‑18, IL‑6, TNF, and CCL2 (Fig. 4J-M), consistent with ongoing inflammasome activation and chronic inflammatory signaling, whereas epithelial *Nlrp3* deficiency significantly reduced these cytokines. IL‑1β levels were low at this time point and did not differ between groups (Fig. 4I). TGFβ concentrations also remained unchanged (Fig. 4H), indicating that epithelial *Nlrp3* regulates neutrophil persistence and inflammatory cytokine production independently of detectable alterations in canonical pro‑fibrotic TGFβ or IL‑1β pathways.

Collectively, these findings demonstrate that alveolar epithelial *Nlrp3* drives the recruitment, activation, and persistence of pro-fibrotic Siglec-F^+^ neutrophils, linking epithelial inflammasome activation to sustained neutrophil‑mediated pathology during silica‑induced lung disease.

### *Nlrp3* deficiency in alveolar epithelial cells reduces early silica-induced lung fibrosis and nodule expansion

**Fig. 5:**
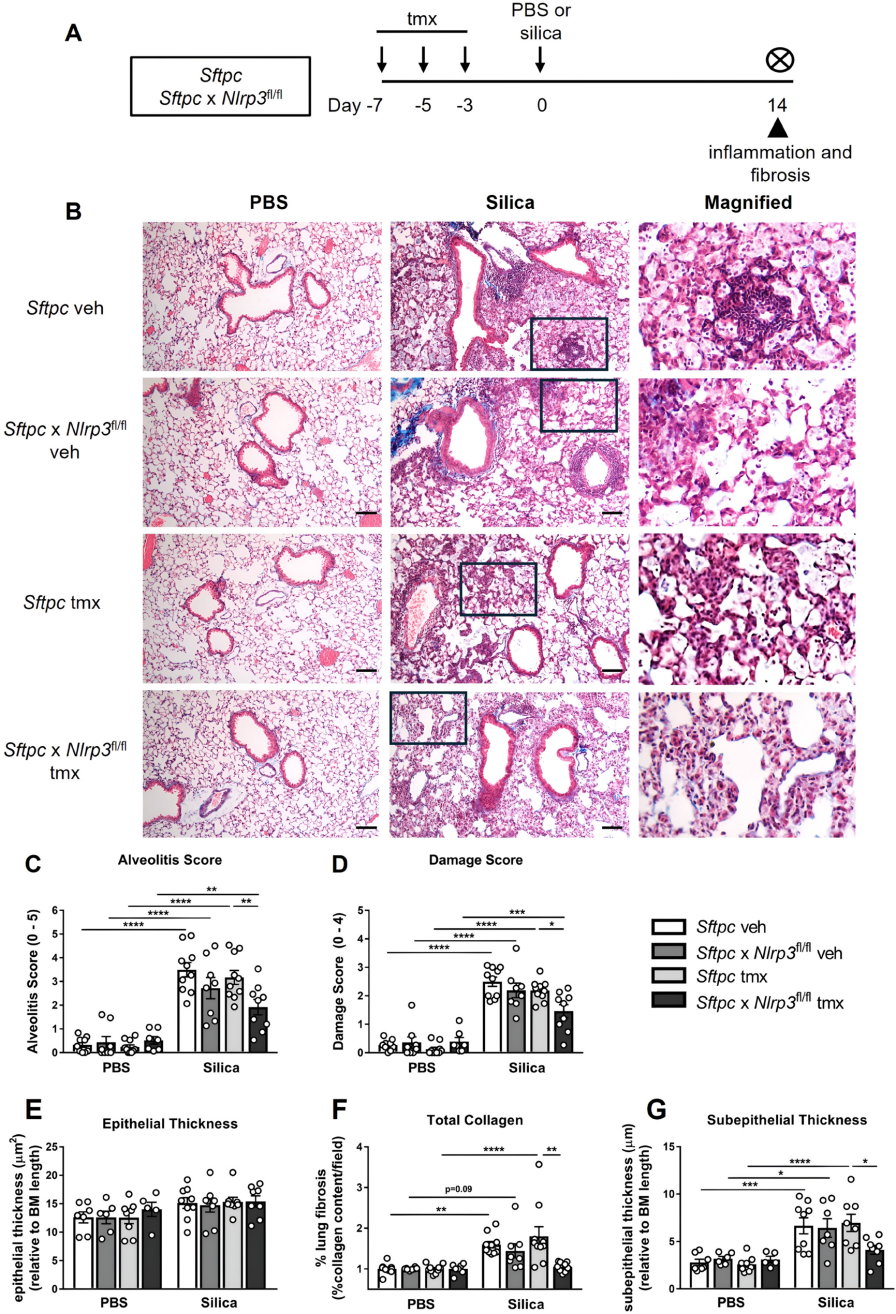
*Nlrp3* deficiency in alveolar epithelial cells reduces early silica-induced lung fibrosis. **A** Experimental timeline for silica-induced lung injury in genetically modified mice. *Sftpc-*CreER^T2^ (*Sftpc*, control) and *Sftpc-*CreER^T2^ x *Nlrp3*^fl/fl^ (*Sftpc* x *Nlrp3*^fl/fl^ - conditional knockout of alveolar epithelial *Nlrp3*) mice received tamoxifen (tmx) or vehicle (veh) on days − 7, -5, and − 3 to induce Cre recombination. Mice were intranasally administered PBS or 2 mg of silica. **B**–**G** On day + 14, lung tissues were formalin-fixed and inflated for histological analysis. **B** Representative images of Masson’s trichrome-stained lung sections at 10x magnification. Scale bar 100 μm. The boxes on these images indicate the areas that are magnified to demonstrate lung tissue damage. Lung sections were randomized, blinded, and scored for **C** alveolitis (scale 0–5) and **D** lung damage (scale 0–4). Quantification of (**E**) epithelial thickness (µm^2^ relative to basement membrane (BM) length), **F** total lung collagen content (Masson’s trichrome pixel intensity per field of view (FOV)), and **G** subepithelial thickness (µm relative to basement-membrane (BM) length). **C**–**G** Data are presented as mean ± SEM, with each data point representing an individual animal. *N* = 7–10 per group. *P < 0.05, **P < 0.01, ***P < 0.001, ****P < 0.0001, One-way ANOVA with Tukey’s multiple comparisons test. Data are pooled from 4 independent experiments

Having established that alveolar epithelial *Nlrp3* drives early IL-18 release and pro-fibrotic neutrophil recruitment, we next assessed whether these inflammatory changes translated into early structural and fibrotic alterations at day 14 post-silica exposure (Fig. 5A, B). To comprehensively assess fibrosis, we combined blinded histopathological scoring, quantitative Masson’s trichrome analysis, morphometric measurements, and silicotic nodule quantification.

Silica-exposed control mice exhibited marked early fibrotic pathology, including increased alveolitis (Fig. 5C), extensive tissue injury (Fig. 5D), and increased collagen deposition throughout both the lung and airways (Fig. 5F, G). These findings were supported by blinded fibrosis scoring and quantitative image analysis demonstrating significant increases in total lung collagen and subepithelial airway collagen accumulation. In contrast, conditional deletion of *Nlrp3* in alveolar epithelial cells significantly attenuated these major fibrotic metrics. *Sftpc* x *Nlrp3*^fl/fl^ mice exhibited reduced alveolitis scores (Fig. 5C) and lung damage scores (Fig. 5D), as well as substantially decreased collagen deposition in both the airway and alveolar compartments (Fig. 5F, G). Epithelial thickness remained unchanged (Fig. 5E), indicating that alveolar epithelial *Nlrp3* deficiency limits early fibrotic remodeling without altering epithelial structural integrity.

Morphometric analysis of trichrome-stained lung sections showed that mean linear intercept (MLI) was unchanged at day 14 (Supplementary Figure S4A, B), consistent with early-stage fibrosis, while alveolar septal thickness, significantly elevated in silica-exposed controls showed a trend toward reduction in alveolar epithelial *Nlrp3*-deficient mice (Supplementary Figure S4C).

**Fig. 6:**
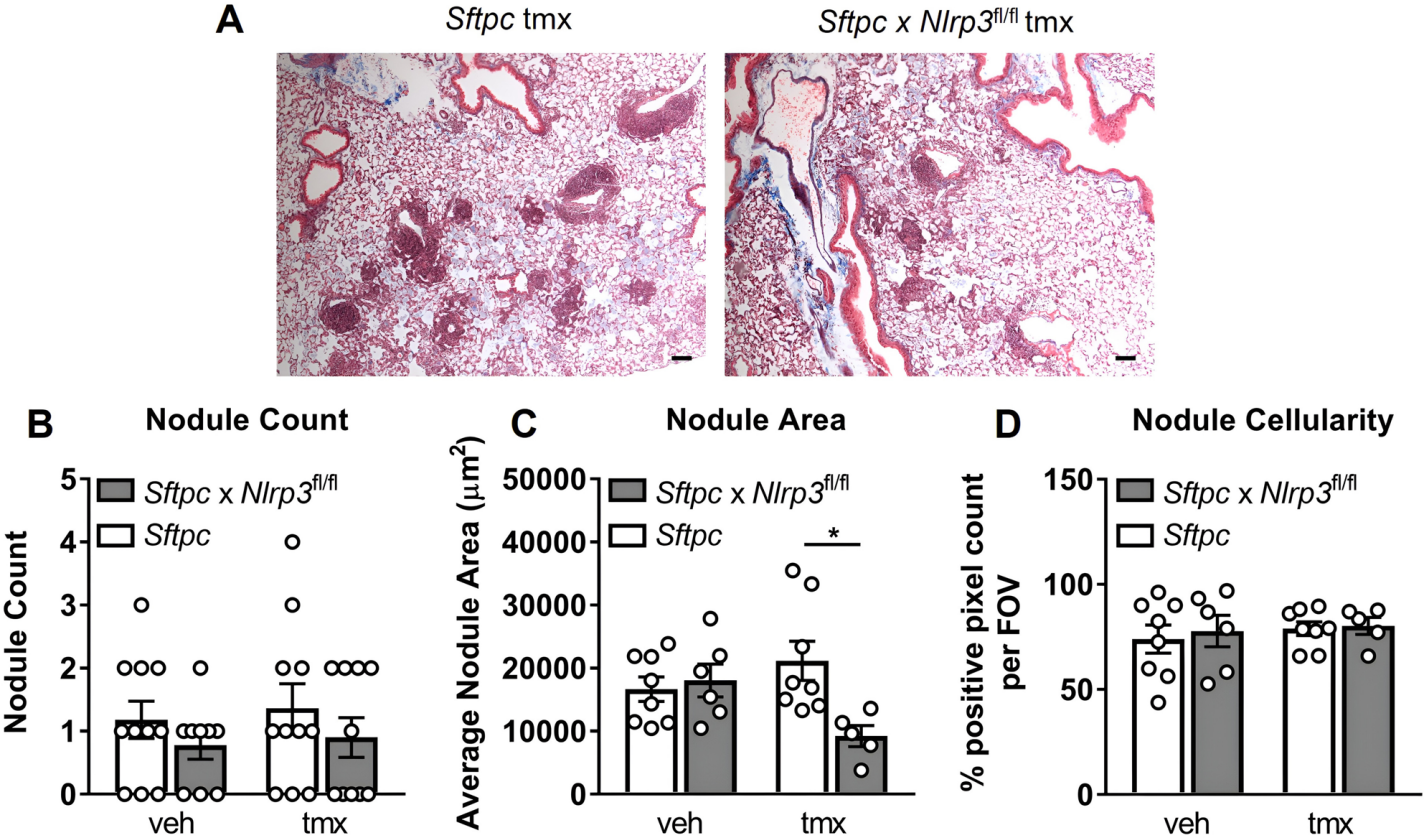
*Nlrp3* deficiency in alveolar epithelial cells reduces early silica-induced nodule expansion. A–D *Sftpc*-CreER^T2^ (*Sftpc*, control) and *Sftpc*-CreER^T2^ x *Nlrp3*^fl/fl^ (*Sftpc* x Nlrp3^fl/fl^, conditional knockout) mice received tamoxifen (tmx) or vehicle (veh) on days − 7, – 5, and − 3 to induce Cre recombination. Mice were subsequently intranasally administered PBS or 2 mg of silica. On day + 14, lung tissues were formalin-fixed and inflated for histological analysis. A Representative images of Masson’s trichrome-stained lung sections at 4x magnification. Scale bar 20 μm. Lung sections were randomized and analyzed blindly for silicotic nodule B count, C average size (µm^2^), and D cellularity (% positive pixel count per FOV). A–D Data are presented as mean ± SEM, with each data point representing an individual animal. N = 5–11 per group. *P < 0.05, One-way ANOVA with Tukey’s multiple comparisons test. Data are pooled from 4 independent experiments

We next examined silicotic nodules, compact collagen-rich lesions formed around silica particles (Fig. 6A, B). Quantitative analysis demonstrated a significant reduction in nodule size in alveolar epithelial *Nlrp3*-deficient mice (Fig. 6C). By contrast, nodule number (Fig. 6B) and cellularity (Fig. 6D) were unchanged, indicating that alveolar epithelial *Nlrp3* primarily regulates the fibrotic expansion rather than the initial formation of nodules.

Together, these comprehensive histological and morphometric analyses demonstrate that alveolar epithelial Nlrp3Nlrp3 is a major driver of early silica-induced fibrotic remodeling, contributing to alveolitis, collagen deposition, septal thickening, and nodule expansion. These findings mechanistically link early epithelial inflammasome activity to the initiation of fibrotic tissue remodelling.

### *Nlrp3* deficiency in alveolar epithelial cells reduces silica-induced α-SMA expression independent of TGFβ

Given that alveolar epithelial *Nlrp3* promotes neutrophil accumulation and early fibrotic remodeling, we next investigated whether these effects extend to myofibroblast activation, a key cellular driver of collagen deposition and disease pathology [34]. Immunohistochemistry was used to assess α-SMA expression, a marker of myofibroblasts, in lung tissue at day 14 post-silica exposure. Silica exposure increased α-SMA expression in both control and *Sftpc*-*Nlrp3*-deficient mice (Supplementary Figure S5A-C). However, α-SMA staining within peribronchial and subepithelial airway regions was significantly reduced in epithelial-deficient mice (Supplementary Figure S5A-C), indicating a localized attenuation of myofibroblast activation in the absence of alveolar epithelial *Nlrp3*.

To assess whether reduced myofibroblast activation reflected altered epithelial integrity or canonical TGFβ signaling, we assessed E-cadherin and TGFβ by immunofluorescence. Both epithelial integrity (E-cadherin) and TGFβ abundance were comparable between genotypes (Supplementary Figure S5D, E), indicating decreased α-SMA expression without detectable changes in TGFβ levels at this timepoint.

Together, these findings indicate that alveolar epithelial *Nlrp3* promotes early myofibroblast activation via a TGFβ‑independent mechanism, consistent with an upstream role for epithelial inflammasome activity and neutrophil‑derived mediators in initiating fibrotic remodeling.

### *Nlrp3* deficiency in *Sftpc*^+^ and *Scgb1a1*^+^ epithelium limits advanced silica-induced pathology

**Fig. 7:**
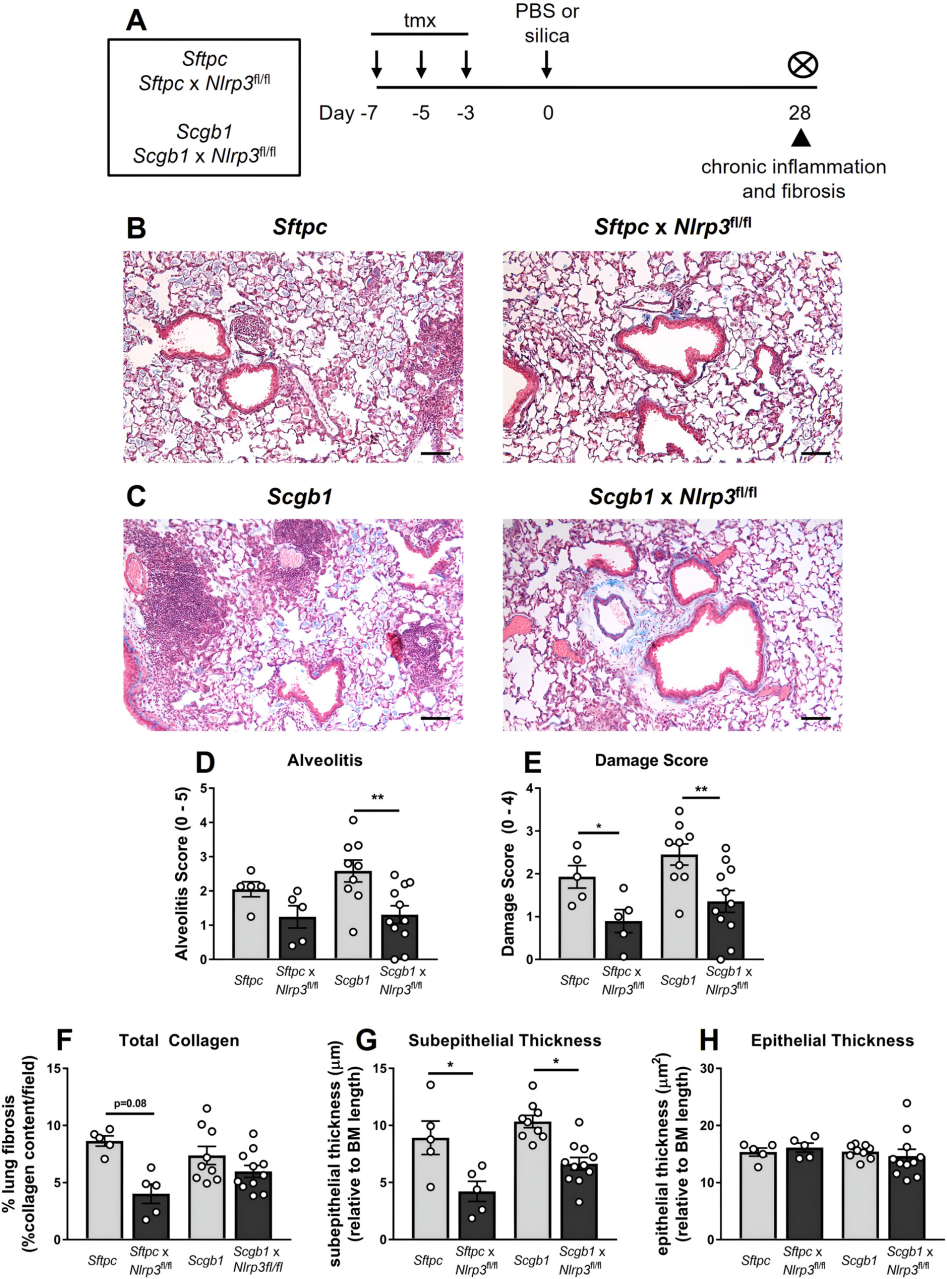
*Nlrp3* deficiency in bronchiolar and alveolar epithelial cells limits advanced silica-induced pathology. **A** Experimental timeline for silica-induced lung injury in genetically modified mice. Two independent Cre models were used: *Sftpc-*CreER^T2^ (*Sftpc*, control) and *Sftpc-*CreER^T2^ x *Nlrp3*^fl/fl^ (*Sftpc* x *Nlrp3*^fl/fl^ – conditional knockout of alveolar epithelial *Nlrp3*) mice, and *Scgb1a1-*CreER™ (*Scgb1*, control) and *Scgb1a1*-CreER™ x *Nlrp3*^fl/fl^ (*Scgb1* x *Nlrp3*^fl/fl^ – conditional knockout of bronchiolar epithelial *Nlrp3*). Mice received tamoxifen (tmx) or vehicle (veh) on days − 7, -5, and − 3 to induce Cre recombination. Mice were intranasally administered PBS or 2 mg of silica. On day + 28, lung tissues were formalin-fixed and inflated. Histological analysis of Masson’s trichrome-stained lung tissue sections was performed. **B**, **C** Representative images at 4x magnification. Lung sections were randomized, blinded, and analyzed or scored for **D** alveolitis (scale 0–5) and **E** lung damage (scale 0–4). Quantification of **F** total lung collagen (Masson’s trichrome staining intensity per field of view (FOV)), **G** subepithelial thickness (µm relative to basement membrane (BM) length), and **H** epithelial thickness (µm^2^ relative to BM length). **B**–**H** Data from each Cre model were collected and analyzed independently. Data are presented as mean ± SEM, with each data point representing an individual animal. *N* = 5–11 per group. *P < 0.05, ** *P* < 0.01, One-way ANOVA with Tukey’s multiple comparisons test. Data are pooled from 2–3 independent experiments

Having established that alveolar epithelial *Nlrp3* drives early inflammatory and fibrotic remodeling through day 14, we next examined whether epithelial *Nlrp3* continues to sustain pathology during the chronic phase. Using parallel conditional knockout models targeting distinct epithelial compartments, we assessed the contribution of *Sftpc*^+^ alveolar and *Scgb1a1*^+^ bronchiolar epithelial deletion of *Nlrp3* to chronic disease at day 28, following the shared experimental timeline shown in Fig. 7A. As expected, bronchiolar epithelial *Nlrp3* deletion partially reduced inflammasome activation at day 3, evidenced by decreased cleaved caspase-1 in lung tissue, and attenuated early airway inflammation, including significant reductions in IMs, Siglec-F⁺ neutrophils, and lower IL-18 levels (Supplementary Figures S6–S7).

We first examined chronic outcomes in the alveolar compartment. Masson’s trichrome staining revealed that alveolar epithelial *Nlrp3* deletion markedly attenuated established silica-induced lung injury at day 28. *Sftpc*-*Nlrp3*-deficient mice exhibited reduced inflammatory cell infiltration and improved preservation of alveolar architecture compared with controls (Fig. 7B). Quantitative scoring confirmed reduced alveolitis (Fig. 7D) and significantly attenuated overall lung damage (Fig. 7E). Accordingly, total lung collagen content was significantly reduced in alveolar epithelial *Nlrp3*-deficient mice (Fig. 7F), accompanied by decreased subepithelial airway collagen deposition (Fig. 7G), while epithelial thickness remained unchanged (Fig. 7H).

**Fig. 8:**
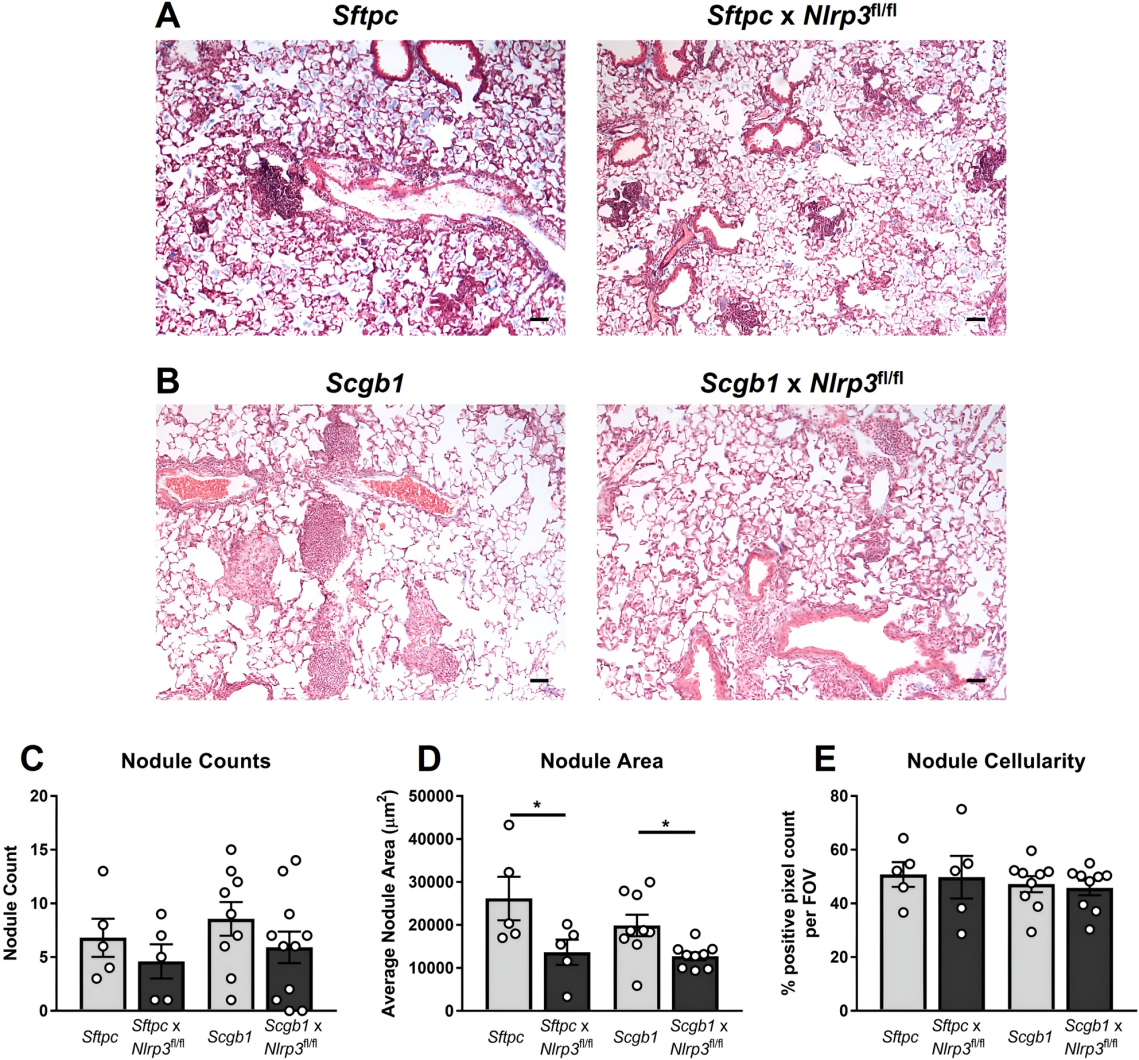
*Nlrp3* deficiency in bronchiolar and alveolar epithelial cells limits advanced silica-induced nodule expansion. *Sftpc-*CreER^T2^ (*Sftpc*, control) and *Sftpc-*CreER^T2^ x *Nlrp3*^fl/fl^ (*Sftpc* x *Nlrp3*^fl/fl^ – tamoxifen-inducible excision of alveolar epithelial *Nlrp3*) mice, and *Scgb1a1-*CreER™ (*Scgb1*, control) and *Scgb1a1*-CreER™ x *Nlrp3*^fl/fl^ (*Scgb1* x *Nlrp3*^fl/fl^ – tamoxifen-inducible excision of bronchiolar epithelial *Nlrp3*) were treated with tamoxifen (tmx) or vehicle (veh) on days − 7, -5, and − 3 to induce Cre recombination. Mice were intranasally administered PBS or 2 mg of silica. On day + 28, lung tissues were formalin-fixed and inflated. **A**, **B** Representative images of Masson’s trichrome-stained sections at 4x magnification. Scale bar 20 μm. Lung sections were randomized and analyzed for nodule **C** count, **D** average size (µm^2^) and **E** cellularity (% positive pixel count per FOV). **A**–**E** Data from each Cre model were collected and analyzed independently. Data are presented as mean ± SEM, with each data point representing an individual animal. *N* = 5–11 per group. *P < 0.05, One-way ANOVA with Tukey’s multiple comparisons test. Data are pooled from 2–3 independent experiments

Quantitative image-based analysis of silicotic nodules demonstrated that alveolar epithelial *Nlrp3* deletion limited fibrotic nodule expansion (Fig. 8A). Although total nodule number and cellularity were unchanged (Fig. 8C, E), *Sftpc*-*Nlrp3*-deficient mice developed significantly smaller nodules, reflected by reduced nodule area (Fig. 8D). These findings indicate that alveolar epithelial *Nlrp3* regulates nodule expansion, not initiation.

We next asked whether bronchiolar epithelial *Nlrp3* similarly contributes to chronic disease. *Scgb1a1*-CreER™ x *Nlrp3*^fl/fl^ mice [20] and *Scgb1a1*-CreER™ controls were analyzed at day 28 (Fig. 7A). Bronchiolar epithelial *Nlrp3* deletion also attenuated established silica-induced lung injury. Lungs from *Scgb1a1*-*Nlrp3*-deficient mice displayed reduced inflammatory infiltration and improved tissue architecture relative to controls (Fig. 7C). Blinded quantitative scoring demonstrated reduced alveolitis (Fig. 7D) and overall injury (Fig. 7E). Total lung collagen content showed a downward trend (Fig. 7F), while subepithelial airway collagen deposition was significantly decreased (Fig. 7G), and epithelial thickness remained unchanged (Fig. 7H).

Analysis of silicotic nodules in the bronchiolar cohort revealed a similar pattern with nodule size, but not nodule number or cellularity significantly reduced (Fig. 8C–E). These data demonstrate that bronchiolar epithelial *Nlrp3*, like alveolar epithelial *Nlrp3*, contributes to fibrotic nodule expansion rather than initiation.

Together, these findings establish that epithelial *Nlrp3* plays a central role in sustaining chronic silica-induced lung pathology. Both alveolar and bronchiolar epithelial compartments contribute to persistent inflammation, collagen deposition, and fibrotic nodule enlargement, identifying epithelial inflammasome activation as a key driver of progressive fibrotic lung disease.

## Discussion

Despite global and local efforts to eliminate silicosis [1, 35], rising case numbers underscore the urgent need for mechanistic insights to guide the development of effective therapies. Our study resolves a longstanding paradox: global *Nlrp3* deficiency protects against silicosis, yet myeloid-specific deletion does not [13, 14], challenging the macrophage-centric paradigm [7, 10]. We identify epithelial cells as the critical source of pathological NLRP3 activity, with epithelial-specific deletion recapitulating the protective effects of global knockout, attenuating caspase-1 activation (Fig. 1), cytokine production (Fig. 2), fibrotic neutrophil recruitment (Figs. 3 and 4), and fibrotic remodeling (Figs. 5, 6, 7 and 8). These findings shift our understanding of inflammasome-driven lung disease.

Our findings align with our prior in vivo reporter mouse study showing that silica induces NLRP3 expression in both alveolar and bronchiolar epithelial cells, indicating that epithelial inflammasome activation occurs across multiple epithelial compartments [13].

The epithelial cell dominance in vivo likely reflects unique biology and differential cytokine storage [28, 36, 37]. While silica activated NLRP3 in both macrophages and epithelial cells in vitro [16], unlike macrophages, which require transcriptional induction of inflammasome substrates, epithelial cells constitutively store IL-18 precursors [28, 36], enabling rapid caspase-dependent release upon activation [38, 39]. Accordingly, epithelial *Nlrp3* deletion selectively impaired IL-18 production (Fig. 2), attenuated recruitment of pro-fibrotic Siglec-F^+^ neutrophils, and lowered neutrophil elastase, a key driver of tissue remodeling [30, 31]. This distinction, masked in global knockout studies [13], establishes IL-18 as a central epithelial output linking inflammasome activation to neutrophil-driven fibrosis, consistent with known roles of IL-1 family cytokines in neutrophil activation and inflammation [40].

In contrast, IL-1β requires transcriptional induction and is predominantly produced by other epithelial subtypes or recruited immune cells [28, 41]. Immunoblot analysis revealed partial reduction of IL-1β in lung tissue, suggesting indirect contributions from alveolar epithelial *Nlrp3*. These findings support a model in which epithelial-derived IL-18 initiates and sustains inflammation, which may then prime infiltrating myeloid cells via NF-κB. Global *Nlrp3* deletion disrupts both arms of this response and confers protection, whereas myeloid-specific deletion leaves the critical epithelial initiation phase intact, offering no protection. Identification of IL-18 as a key epithelial output aligns with its role in shaping neutrophil responses, particularly recruitment of Siglec-F^+^ neutrophils (Fig. 3), a pro-fibrotic subset producing TGFβ, collagen I, and α-SMA [13, 29]. Our global *Nlrp3* deletion study linked these cells to disease severity [13], and Barry et al. demonstrated that myeloid *Nlrp3* does not regulate their recruitment [14]. The present study advances this understanding by implicating epithelial-derived IL-18 as an upstream driver, providing a mechanistic link between epithelial *Nlrp3* and neutrophil-driven fibrosis. Consistent with this model, epithelial *Nlrp3* deletion reduced Siglec-F^+^ neutrophils (Figs. 3 and 4), supporting a non-redundant role for epithelial inflammasome signaling in orchestrating neutrophil activity.

In line with their proposed pathogenic role, Siglec-F⁺ neutrophils exhibited a pro-fibrotic transcriptional profile, including elevated expression of *Tgfb1*, *Fgf2*, *Tnf*, and *Il1b*, alongside expression of inflammasome-associated receptors such as *Il18rap* and *Il1r2* (Fig. 3). This profile suggests that these cells may be equipped to respond to epithelial-derived IL-18 at the transcriptional level and are primed to promote tissue remodeling. The concurrent expression of *Il1r2*, which encodes a decoy receptor for IL-1, further raises the possibility that these cells may selectively modulate IL-1 signaling. This could potentially favor sustained, pro-fibrotic activity over acute inflammatory responses.

Epithelial *Nlrp3* not only promoted the initial recruitment of pro-fibrotic neutrophils but also their sustained accumulation. Siglec-F⁺ neutrophils accounted for approximately 50% of total neutrophils at day 14, and their sustained accumulation was associated with epithelial IL‑18, consistent with the marked reduction in both Siglec‑F⁺ neutrophils and IL‑18 in epithelial *Nlrp3*‑deficient mice. Additionally, alveolar epithelial *Nlrp3* deletion was accompanied by attenuation of IL-18, IL-6, TNF, and CCL2, consistent with a self-amplifying epithelial inflammasome signal that perpetuates neutrophil-driven inflammation into the chronic phase (Fig. 4). The absence of IL-1β elevation at day 14, and the unchanged TGFβ across groups, further reinforces that this sustained inflammatory circuit operates through IL-18 rather than canonical IL-1β or TGFβ pathways.

While additional epithelial factors (DAMPs, pyroptotic contents) may amplify these responses, the failure of myeloid NLRP3 to compensate underscores epithelial dominance. Recent complementary evidence further shows that Siglec-F^+^ neutrophils promote fibrosis in the bleomycin-induced lung injury model [42]. In this setting, vasoactive intestinal peptide production (VIP) by AMs in the absence of TRPV1^+^ nociceptors elevated TGFβ1, which together with VIP signalling, promotes the accumulation of Siglec-F^+^ neutrophils and NET release. Together, these findings support a conserved pro-fibrotic role for Siglec-F⁺ neutrophils across lung injury models, positioning epithelial NLRP3 as a key upstream regulator of their recruitment in silicosis. While IL-18 knockout studies in silicosis are lacking, recent work shows that fibroblast-derived IL-18 contributes to immune remodeling and fibrosis [43]. Although this mechanism differs from epithelial IL-18 signaling, it reinforces the concept that IL-18 is a pro-fibrotic mediator in silicosis. Notably, epithelial and fibroblast-derived IL-18 may represent temporally distinct but complementary signals, with epithelial NLRP3-dependent IL-18 driving early inflammasome-initiated responses, and fibroblast-derived IL-18 potentially amplifying the inflammatory milieu during established fibrosis.

Epithelial *Nlrp3* deletion also reduced α-SMA expression within peribronchial and subepithelial regions, independent of TGFβ (Supplementary Figure S5), indicating a non-canonical fibrotic pathway. This contrasts with global deletion, where both TGFβ and α-SMA decreased [11], suggesting distinct upstream mechanisms. Reduced neutrophil elastase may limit elastase-mediated fibroblast activation [44], while decreased epithelial pyroptosis would lower DAMP release. This TGFβ-independent mechanism may explain how epithelial-targeted therapies could bypass resistance pathways that limit current anti-fibrotic approaches.

Comparable protection from alveolar (*Sftpc*^+^) and bronchiolar (*Scgb1a1*^+^) epithelial *Nlrp3* deletion at day 28 (Figs. 7 and 8) is consistent with early reductions in caspase‑1 activation, Siglec‑F⁺ neutrophils, and IL‑18 observed in the bronchiolar compartment (Supplementary Figures S6-S7), indicating that both epithelial regions contribute to sustaining chronic pathology. This reflects silica particle penetration throughout airways and alveoli [13], universal epithelial *Nlrp3* expression, and parallel inflammatory cascades. Therapeutically, these findings highlight the need for epithelial-targeted NLRP3 inhibition to reach both airway and alveolar regions, supporting the rationale for inhaled drug delivery approaches capable of accessing the full epithelial surface. Notably, epithelial deletion reduced nodule size without affecting formation or cellularity (Figs. 6 and 8), revealing that initiation is NLRP3-independent (requiring macrophage recruitment and phagocytosis) while expansion is epithelial NLRP3-dependent (requiring sustained IL-18-driven inflammation).

These findings suggest a potential shift in therapeutic strategy. Epithelial-targeted NLRP3 inhibition delivered by inhalation may offer advantages over systemic approaches, including direct delivery to disease sites, preservation of systemic immune function, and targeting of TGFβ-independent pathways that may contribute to resistance against current therapies. Unlike global inhibition, which carries risks of systemic immunosuppression, and myeloid-specific targeting, which has shown limited efficacy, epithelial-directed approaches may interrupt early initiating signals. The findings, showing that epithelial NLRP3 drives nodule expansion but not formation, further indicate that therapeutic windows may exist for both prevention in high-risk exposure settings and for limiting progression in established disease. Whether IL-18 neutralization alone can achieve similar benefit to NLRP3 inhibition remains to be determined, as our findings suggest epithelial pyroptosis and DAMP release also play contributory roles.

Several limitations merit consideration. Our single-dose instillation model does not fully replicate chronic occupational exposure patterns; repeated low-dose exposure models may better capture the cumulative inflammatory and fibrotic dynamics seen in occupational silicosis. Second, Cre-lox efficiency (~ 60–80%) could explain incomplete protection. While our findings implicate IL-18 as a key epithelial output, additional factors, including DAMPs and pyroptotic contents, likely contribute to neutrophil-driven fibrosis and warrant mechanistic dissection. Lastly, multiple attempts in the current and previous studies [13, 14] to detect NLRP3 protein expression in the lung via immunofluorescence were unsuccessful. NLRP3 could not be reliably visualized in lung epithelial cells, and substantial background staining was observed in *Nlrp3*^−/−^ tissue, underscoring the low specificity of currently available antibodies. Future studies should employ repeated exposure models, single-cell resolution of NLRP3^+^ epithelial subtypes, and genetic or pharmacologic IL-18 blockade. Comparative analysis of bronchiolar versus alveolar epithelial targeting during early versus established disease will be critical for developing therapeutic strategies.

Taken together, this study establishes epithelial cells as the primary source of pathogenic NLRP3 activity in silicosis, resolving a longstanding paradox and defining a new paradigm in silica-driven pulmonary disease. Epithelial-derived IL-18 initiates and sustains persistent pro-fibrotic neutrophil recruitment and amplifies inflammatory cascades independently of canonical TGFβ pathways, with *Nlrp3* deficiency conferring protection across both alveolar and bronchiolar compartments from acute inflammation through to established fibrosis. This work demonstrates how cell-type-specific approaches can resolve mechanistic questions and transform therapeutic strategy from broad pathway inhibition to precision targeting of pathogenic cell populations.

## Electronic Supplementary Material

Below is the link to the electronic supplementary material.

Supplementary Material 1.

## Data Availability

The data that support the findings of this study are available from the corresponding author upon reasonable request.

## Abbreviations

α-SMA: Alpha smooth muscle actin
BAL: Bronchoalveolar lavage fluid
CCL2: C-C motif chemokine ligand 2
CreER: Cre recombinase-estrogen receptor fusion protein
DAMP: Danger associated molecular pattern
ELISA: Enzyme linked immunosorbent assay
FBS: Fetal bovine serum
IFNγ: Interferon gamma
IL-1β: Interleukin-1 beta
IL1r2: Interleukin-1 receptor 2
IL-6: Interleukin-6
IL-10: Interleukin-10
IL-12p20: Interleukin-12p20
IL-18: Interleukin-18
Il18ra: Interleukin-18 receptor accessory protein
MCP-1: Monocyte chemoattractant protein-1
MLI: Mean linear intercept
NE: Neutrophil elastase
NLRP3: NLR family pyrin domain containing 3
*Nlrp3*^fl/fl^: *Nlrp3* floxed/floxed
PBS: Phosphate buffered saline
TGFβ: Transforming growth factor beta
TNF: Tumor necrosis factor
Scgb1a1: Secretoglobin family 1 A member 1
Sftpc: Surfactant protein C
Tmx: Tamoxifen
Veh: Vehicle
