# Supplementary material for: Epithelial NLRP3 drives silica-induced lung injury and fibrosis through IL-18 and pro-fibrotic neutrophil recruitment
Source: Part Fibre Toxicol. 2026 May 3;23:30. doi: 10.1186/s12989-026-00682-9 (PMC13214385; doi:10.1186/s12989-026-00682-9)

SUPPLEMENTARY MATERIAL

Epithelial NLRP3 drives silica-induced lung injury and fibrosis through IL-18 and pro-fibrotic neutrophil recruitment

Maggie Lam[1,2], Kristian T Barry[1,2], Christopher J Hodges[1,2], Alison C West[1,2], Christopher M Harpur[1,2], Ashley Mansell[1,2,3], and Michelle D Tate[1,2#]

1. Centre for Innate Immunity and Infectious Disease, Hudson Institute of Medical Research, Clayton, Victoria, 3168, Australia.
2. Department of Molecular Translational Sciences, Monash University, Clayton, Victoria, 3186, Australia.
3. Department of Microbiology, Anatomy, Physiology, and Pharmacology, La Trobe University, Bundoora, Victoria, 3086, Australia.

#Correspondence: Michelle D. Tate; Address: Centre for Innate Immunity and Infectious Diseases, Hudson Institute of Medical Research, 27-31 Wright Street, Clayton, Victoria, 3168, Australia. Email: michelle.tate@hudson.org.au.

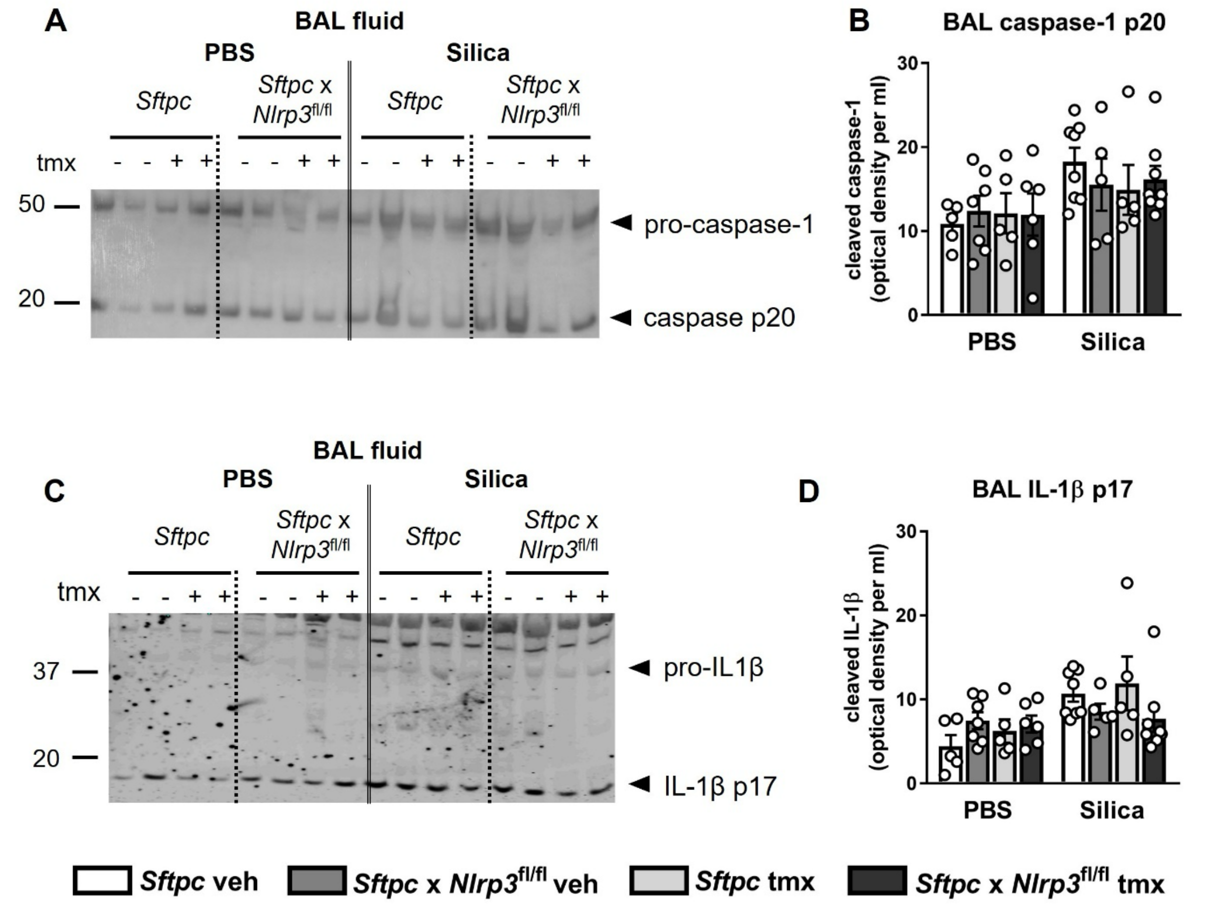

Supplementary Figure S1. Alveolar epithelial-NLRP3 contributes to silica-induced inflammasome activation. (A-D) *Sftpc*-CreER^{T2} (*Sftpc*, control) and *Sftpc*-CreER^{T2} x *Nlrp3*$^{fl/fl}$ (*Sftpc* x *Nlrp3*$^{fl/fl}$, conditional knockout) mice received tamoxifen (tmx) or vehicle (veh) on days -7, -5, and -3 to induce Cre recombination. Mice were subsequently intranasally administered PBS or 2 mg of silica on day 0. Analysis was performed on day +3. Immunoblot of equivalent volume of concentrated BAL fluid for (A) caspase-1 (p20) and (B) IL-1β (p17). Protein expression of (C) caspase-1 (p20) and (D) IL-1β (p17) represented as optical density per ml. Data are presented as mean ± SEM, with each data point representing an individual animal. N=5-8 per group. One-way ANOVA with Tukey's multiple comparisons test. No statistically significant differences were detected across groups. Data are pooled from 3 independent experiments.

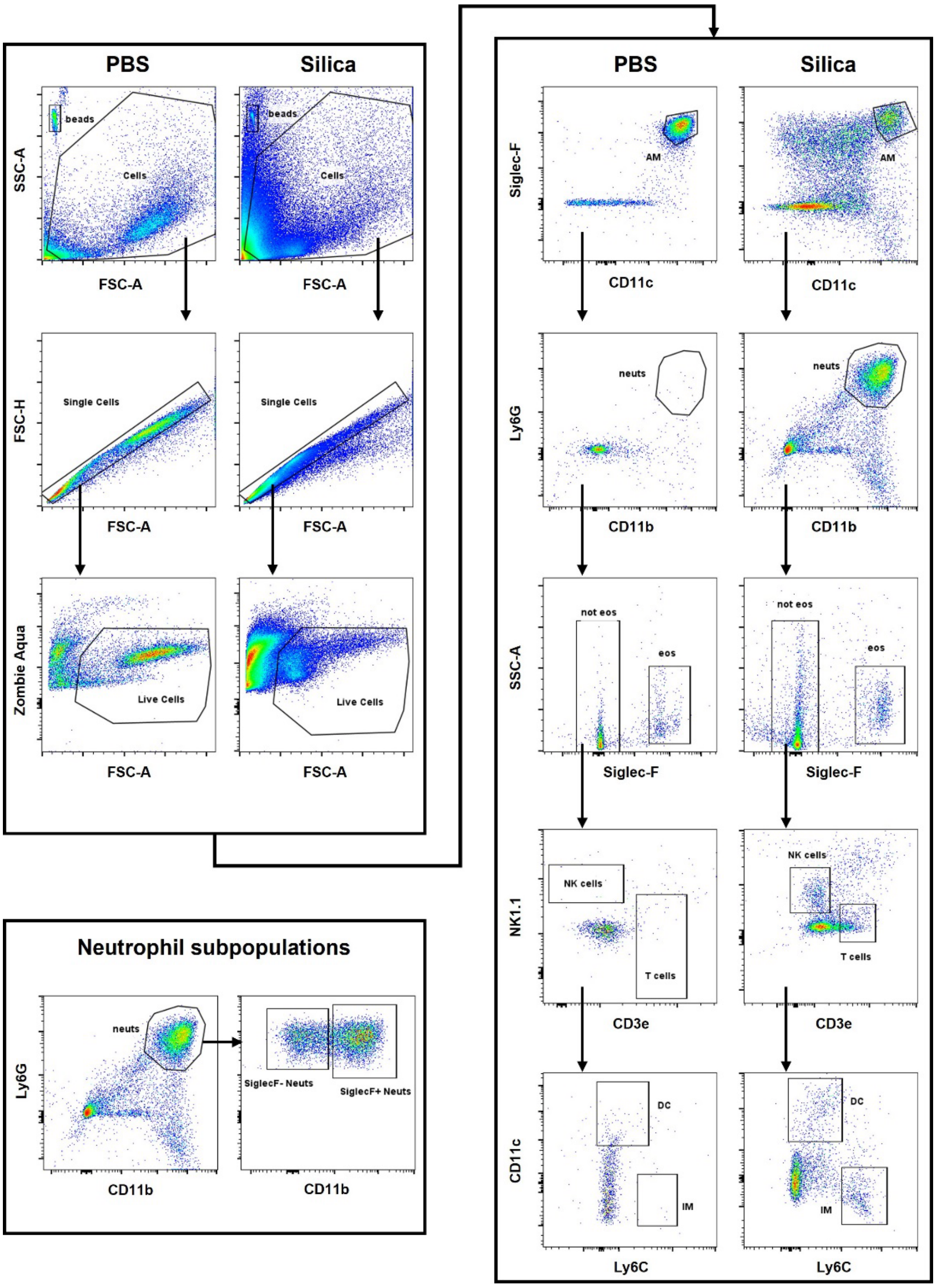

Supplementary Figure S2. Flow cytometry gating strategy for analysis of BAL cells. (A-D) *Sftpc*-CreER^{T2} (*Sftpc*, control) and *Sftpc*-CreER^{T2} x *Nlrp3*$^{fl/fl}$ (*Sftpc* x *Nlrp3*$^{fl/fl}$, conditional knockout) mice received tamoxifen (tmx) or vehicle (veh) on days -7, -5, and -3 to induce Cre recombination. Mice were subsequently intranasally administered PBS or 2 mg of silica on day 0. Flow cytometric analysis on BAL cells was performed on day +3. Representative flow cytometry gating strategy for live (Zombie Aqua^{-}) alveolar macrophages (AM), neutrophils

(neuts), neutrophil subpopulations, eosinophils (eos), NK cells, T cells, inflammatory monocytes/macrophages (IMs) and dendritic cells (DC). Calibration beads were utilized to enumerate cells.

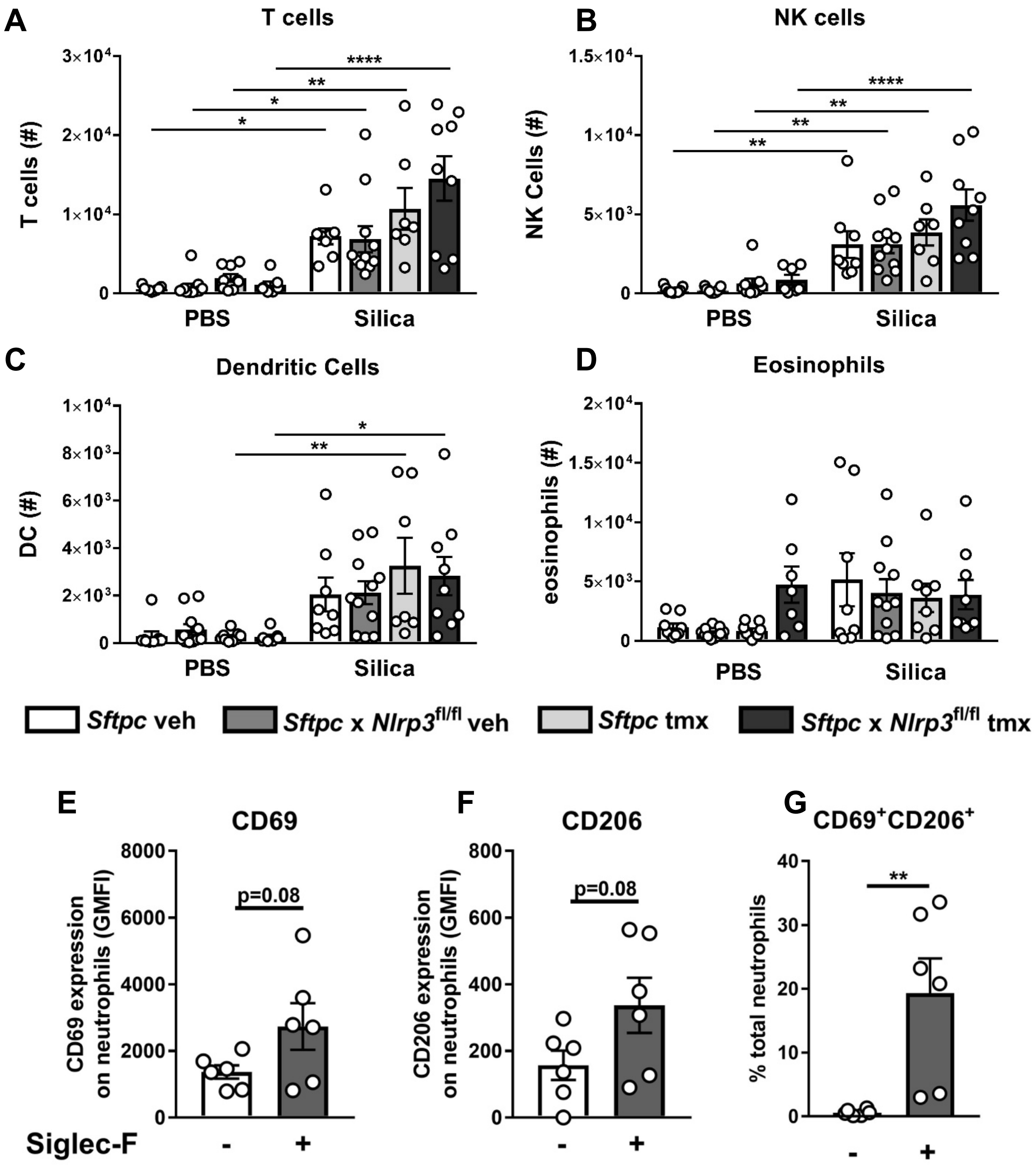

Supplementary Figure S3. NLRP3 deficiency in alveolar epithelial cells does not alter T cell, natural killer cell, dendritic cell, and eosinophil numbers in the airways. (A-D) *Sftpc*-CreER^{T2} (*Sftpc*, control) and *Sftpc*-CreER^{T2} x *Nlrp3*$^{fl/fl}$ (*Sftpc* x *Nlrp3*$^{fl/fl}$, conditional knockout) mice received tamoxifen (tmx) or vehicle (veh) on days -7, -5, and -3 to induce Cre

recombination. Mice were subsequently intranasally administered PBS or 2 mg of silica on day 0. Numbers (#) of live (A) conventional T cells, (B) natural killer cells (NK cells), (C) dendritic cells (DC), and (D) eosinophils in BAL on day +3, determined by flow cytometry. Data are presented as mean ± SEM, with each data point representing an individual animal. N=5-8 per group. *P<0.05, **P<0.01, ****P<0.0001, One-way ANOVA with Tukey's multiple comparisons test. Data are pooled from 3 independent experiments. (E-G) Surface expression of CD69 and CD206 on Siglec-F$^-$ and Siglec-F$^+$ neutrophil subsets, presented as geometric mean fluorescence intensity (GMFI) or % of total neutrophils. Each data point represents an individual animal. N= 6, **P < 0.01, Student's t test.

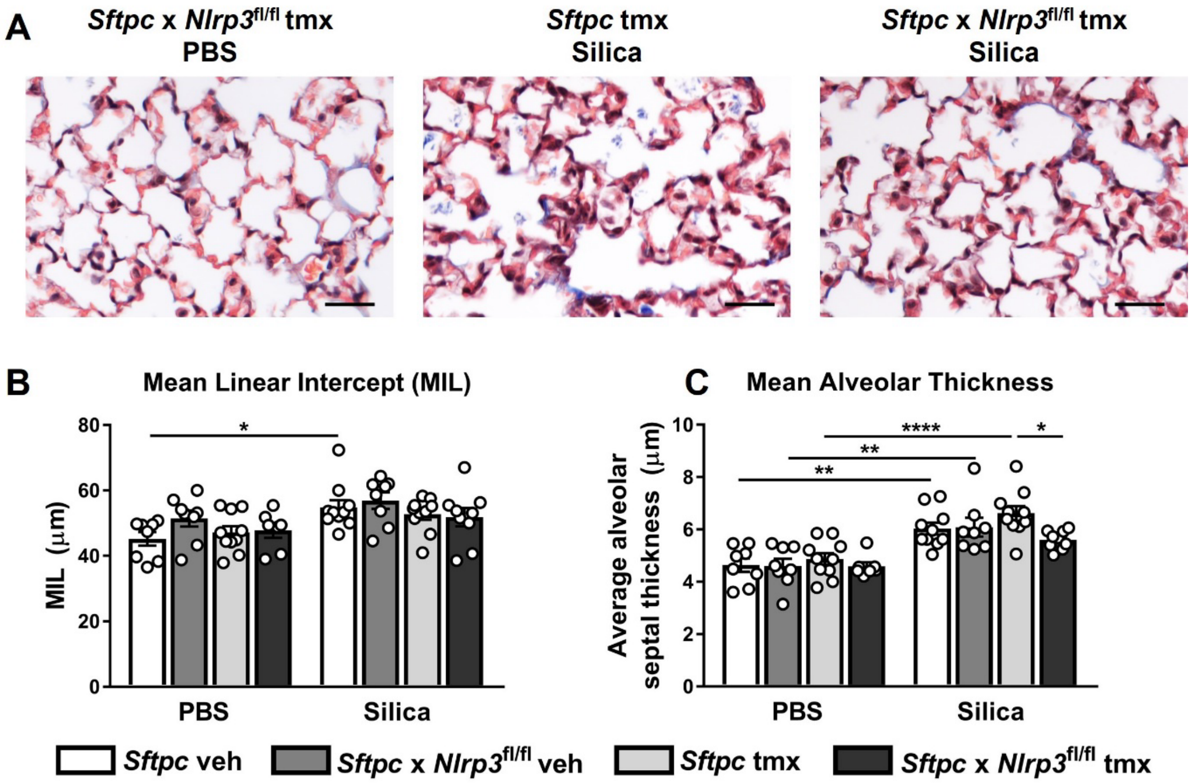

Supplementary Figure S4. NLRP3 deficiency in alveolar epithelial cells reduces early silica-induced lung fibrosis. (A-C) *Sftpc*-CreER^{T2} (*Sftpc*, control) and *Sftpc*-CreER^{T2} x *Nlrp3*$^{fl/fl}$ (*Sftpc* x *Nlrp3*$^{fl/fl}$, conditional knockout) mice received tamoxifen (tmx) or vehicle (veh) on days -7, -5, and -3 to induce Cre recombination. Mice were subsequently intranasally administered PBS or 2 mg of silica on day 0. On day +14, lung tissues were formalin-fixed and inflated. Histological analysis of Masson's trichrome-stained lung tissue sections was performed. (A) Representative images at 10x magnification. Scale bar 100 μm. Lung sections were randomized, blinded, and analyzed for the (B) average distance between alveolar septa within the lung (mean linear intercept, MLI) and mean alveolar thickness (μm). N=7-10. *P<0.05, **P<0.01, ****P<0.0001, One-way ANOVA with Tukey's multiple comparisons test. Data are pooled from 4 independent experiments.

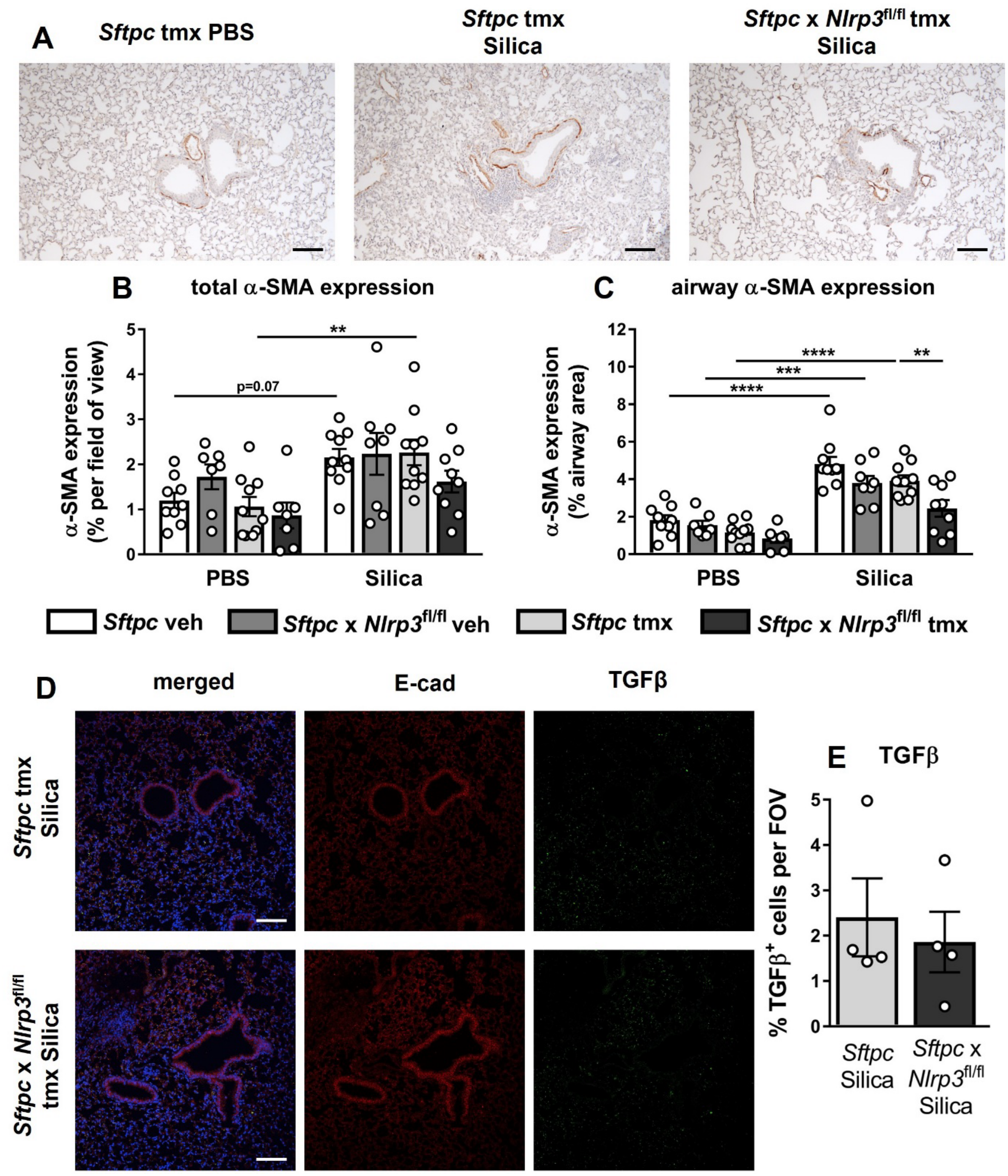

Supplementary Figure S5. NLRP3 deficiency in alveolar epithelial cells reduces silica-induced α-SMA expression independent of TGFβ. (A-G) *Sftpc*-CreER^{T2} (*Sftpc*, control) and *Sftpc*-CreER^{T2} x *Nlrp3*$^{fl/fl}$ (*Sftpc* x *Nlrp3*$^{fl/fl}$, conditional knockout) mice received tamoxifen (tmx) or vehicle (veh) on days -7, -5, and -3 to induce Cre recombination. Mice were subsequently intranasally administered PBS or 2 mg of silica on day 0. On day +14, lung tissues

were formalin-fixed and inflated. (A) Representative images at 10x magnification of immunohistochemical analysis of α-SMA in lung tissue sections. Scale bar 100μm. Quantification of (B) total and (C) airway α-SMA expression determined with ImageJ software. Data are presented as % positive pixel count per FOV. (D) Representative images at 10x magnification of immunofluorescence analysis of TGFβ and E-cadherin in lung sections. Scale bar 100μm. (E) Quantification of TGFβ expression determined with ImageJ software. Data are presented as %$TGF\beta^+$ cells per FOV. (B, C, E) Data are presented as mean ± SEM, with each data point representing an individual animal. N=4-10 per group. *P<0.05, One-way ANOVA with Tukey's multiple comparisons test. Data are pooled from 4 independent experiments.

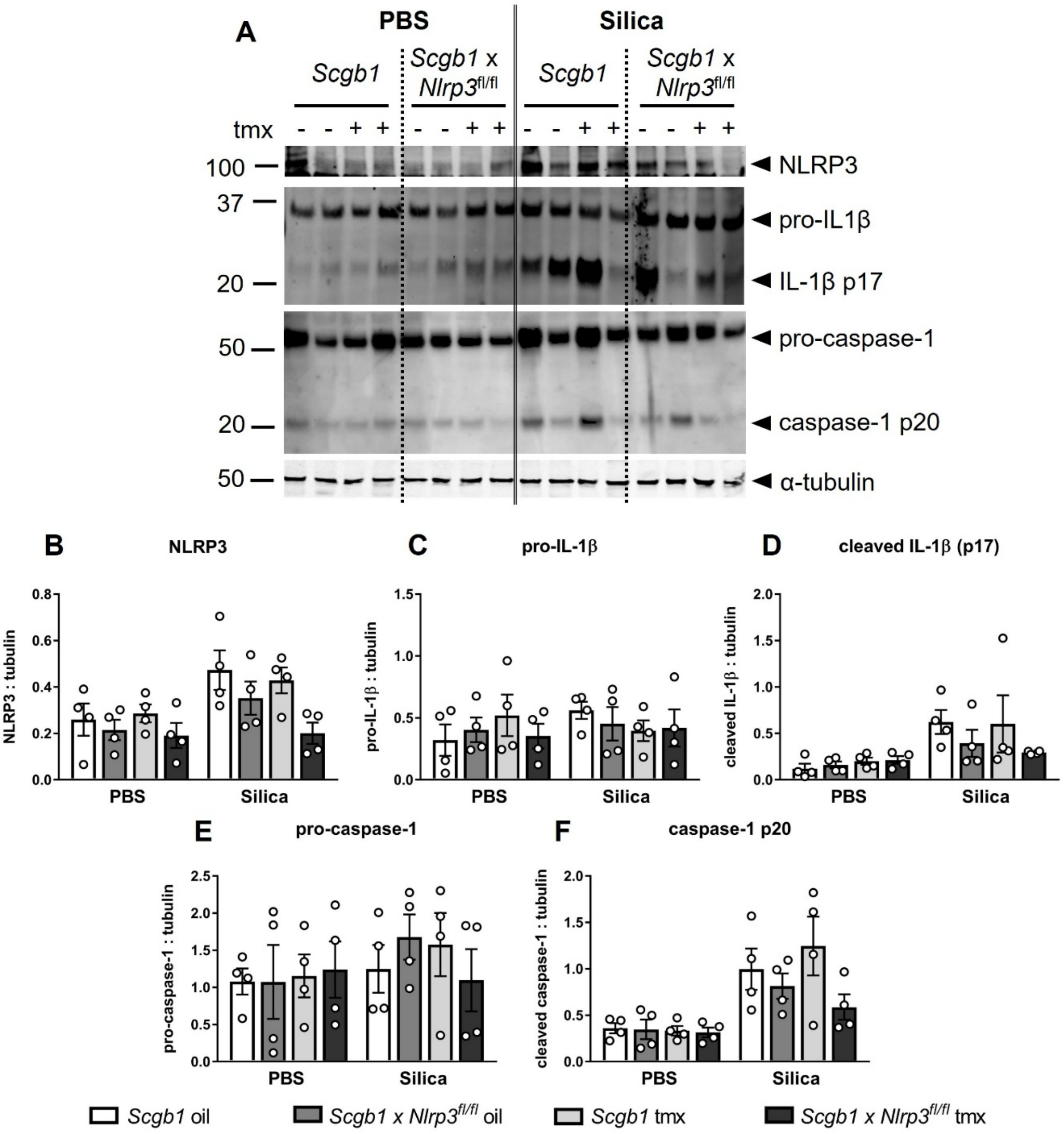

Supplementary Figure S6. Bronchiolar epithelial-NLRP3 contributes to silica-induced inflammasome activation. (A-F) *Scgb1a1*-CreER™ (*Scgb1*, control) and *Scgb1a1*-CreER™ x *Nlrp3*$^{fl/fl}$ (*Scgb1 x Nlrp3*$^{fl/fl}$, conditional knockout) mice received tamoxifen (tmx) or vehicle (veh) on days -7, -5, and -3 to induce Cre recombination. Mice were subsequently intranasally administered PBS or 2 mg of silica on day 0. Analysis was performed on day +3. Immunoblot of NLRP3, pro-IL1β, IL-1β (p17), pro-caspase-1 (p45), cleaved caspase-1 (p20), and tubulin proteins in lung lysates collected at day 3. Expression of (B) NLRP3, (C) pro-IL1β, (D) cleaved IL-1β (p17), (E) pro-caspase-1 (p45), and (F) cleaved caspase-1 (p20) relative to tubulin. (B-

F) Data are presented as mean ± SEM, with each data point representing an individual animal. N=4 per group. One-way ANOVA with Tukey's multiple comparisons test. Data are pooled from 2 independent experiments.

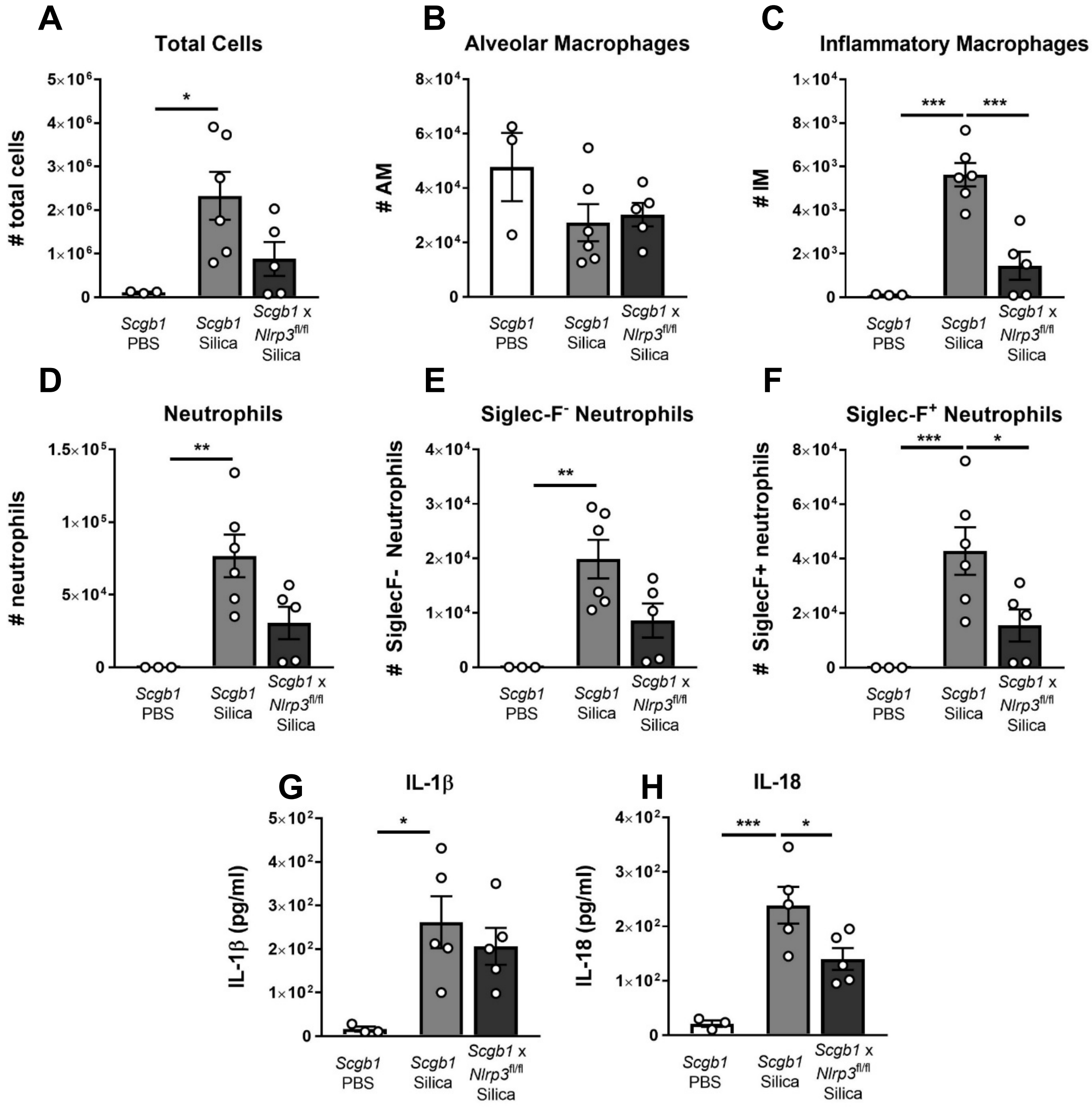

Supplementary Figure S7. NLRP3 deficiency in bronchiolar epithelial cells limits infiltration of fibrotic neutrophils following silica exposure. (A-H) *Scgb1a1*-CreER^{T2} (*Scgb1*, control) and *Scgb1a1*-CreER^{T2} x *Nlrp3*$^{fl/fl}$ (*Scgb1a1*x *Nlrp3*$^{fl/fl}$, conditional knockout) mice received tamoxifen (tmx) or vehicle (veh) on days -7, -5, and -3 to induce Cre recombination. Mice were subsequently intranasally administered PBS or 2 mg of silica. Analysis was performed on day +3. Numbers (#) of live (A) total live cells, (B) alveolar macrophages (AMs), (C) Ly6C^{hi} inflammatory macrophages (IMs), and (D) Ly6G^{+} neutrophils

in BAL, determined by flow cytometry. Numbers (#) of live (E) Siglec-F^- and (F) Siglec-F^+ neutrophils. Levels of (G) IL-1β and (H) IL-18 in BAL fluid, determined by ELISA. (A-H) Data are presented as mean ± SEM, with each data point representing an individual animal. N=3-5 per group. *$P<0.05$, **$P<0.01$, ***$P<0.001$, One-way ANOVA with Tukey's multiple comparisons test.

Figure 1B- Full Blots

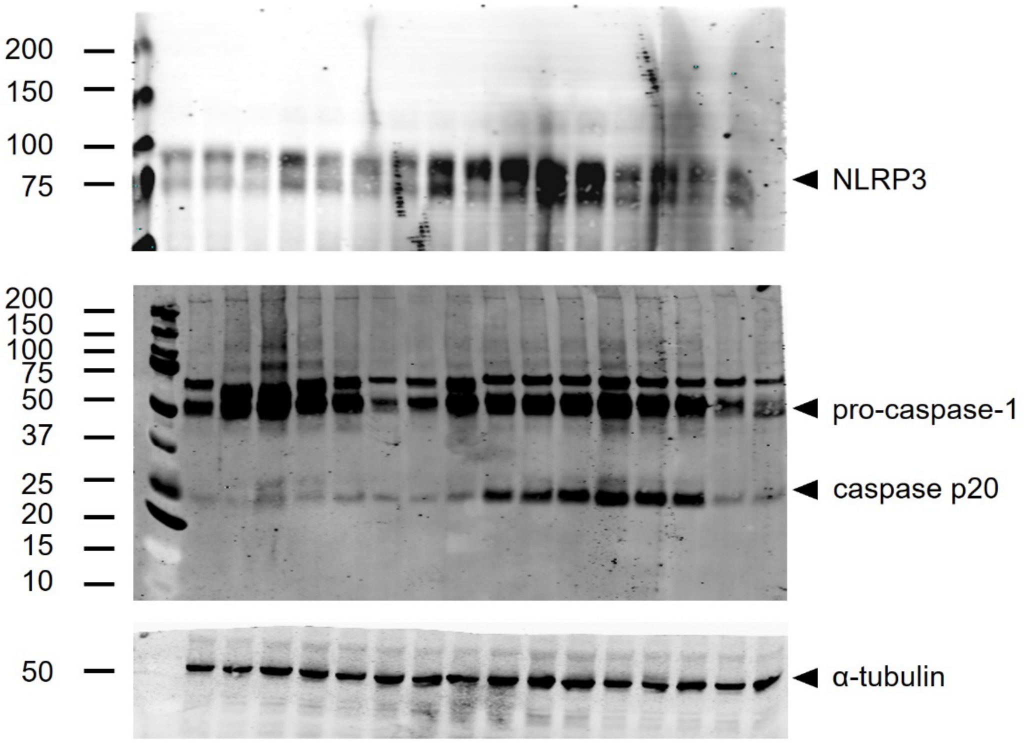

Supplementary Figure 1A- Full Blot

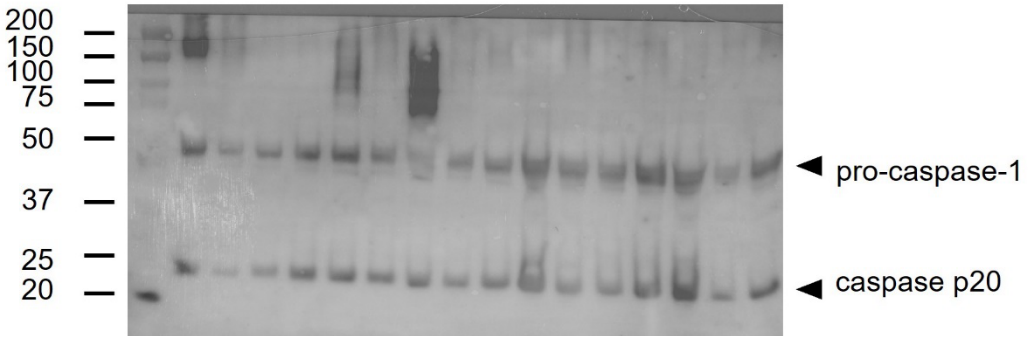

Figure 2F- Full Blots

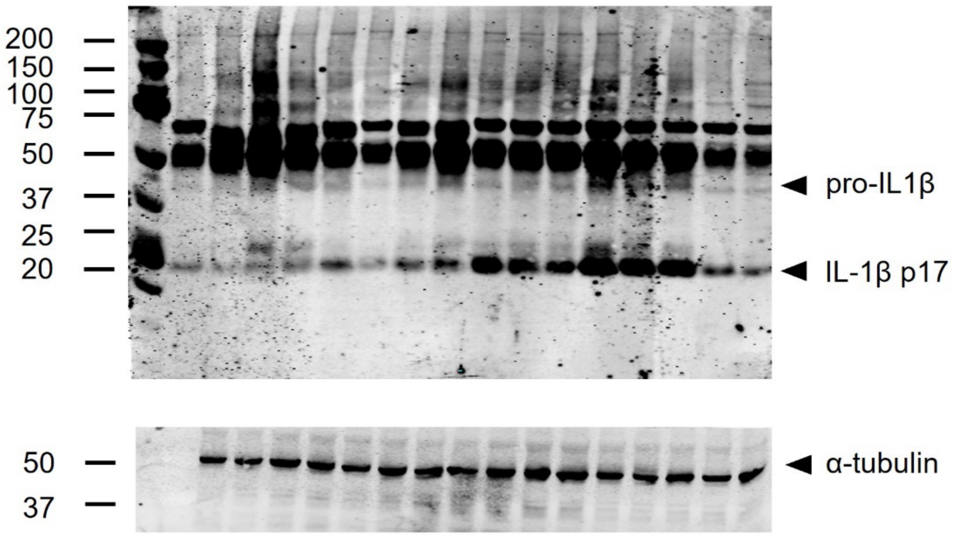

Supplementary Figure 1C- Full Blots

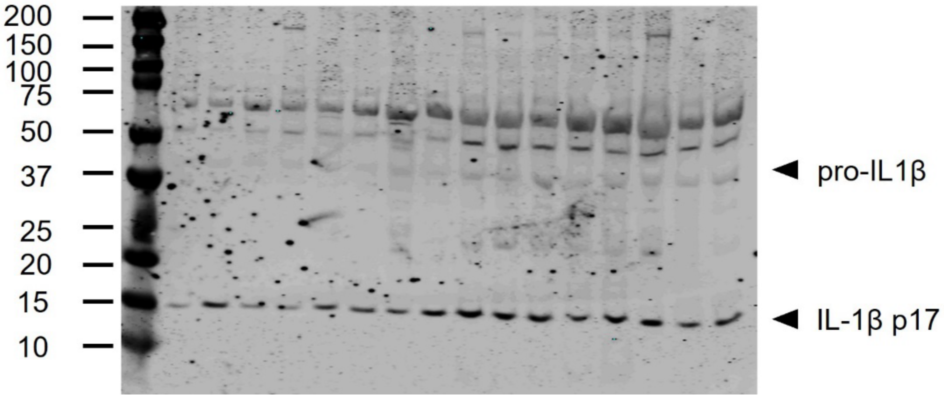

Supplementary Figure 7- Full Blots

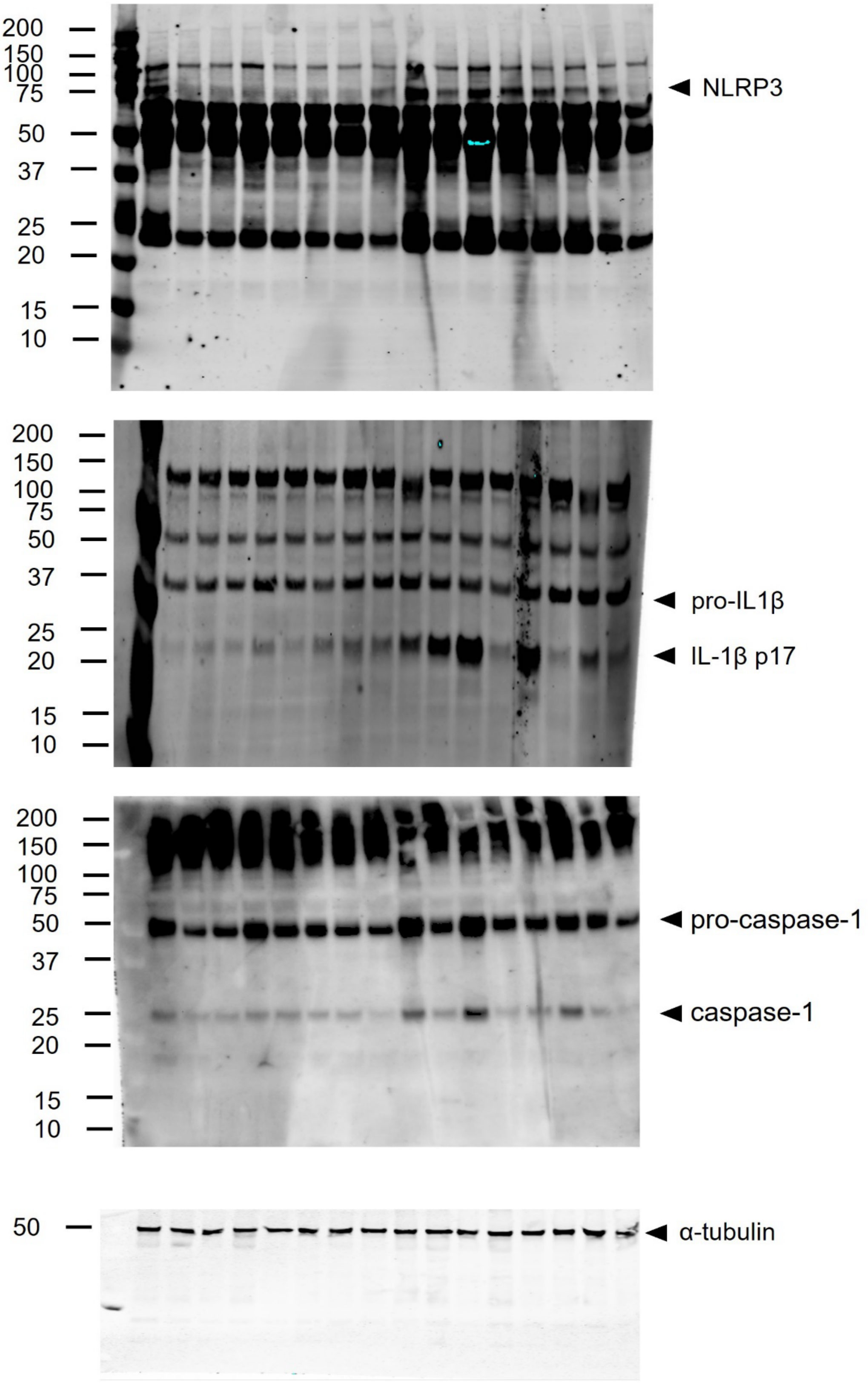

Supplement: Supplementary file 1 — Supplementary Material 1. [file 12989_2026_682_MOESM1_ESM.pdf]
